# Erythrocyte microRNAs show biomarker potential and implicate multiple sclerosis susceptibility genes

**DOI:** 10.1002/ctm2.22

**Published:** 2020-04-10

**Authors:** Kira Groen, Vicki E. Maltby, Rodney J. Scott, Lotti Tajouri, Jeannette Lechner‐Scott

**Affiliations:** ^1^ School of Medicine and Public Health University of Newcastle Callaghan New South Wales Australia; ^2^ Centre for Brain and Mental Health Research Hunter Medical Research Institute New Lambton Heights New South Wales Australia; ^3^ Department of Neurology John Hunter Hospital New Lambton Heights New South Wales Australia; ^4^ Cancer Hunter Medical Research Institute New Lambton Heights New South Wales Australia; ^5^ Division of Molecular Medicine Pathology North John Hunter Hospital New Lambton Heights New South Wales Australia; ^6^ School of Biomedical Sciences and Pharmacy University of Newcastle Callaghan New South Wales Australia; ^7^ Faculty of Health Sciences and Medicine Bond University Robina Queensland Australia; ^8^ Dubai Police Scientific Council Dubai United Arab Emirates

**Keywords:** biomarker, erythrocytes, extracellular vesicles, microRNA, multiple sclerosis, next‐generation sequencing

## Abstract

**Background:**

Multiple sclerosis is a demyelinating autoimmune disease, for which there is no blood‐borne biomarker. Erythrocytes may provide a source of such biomarkers as they contain microRNAs. MicroRNAs regulate protein translation through complementary binding to messenger RNA. As erythrocytes are transcriptionally inactive, their microRNA profiles may be less susceptible to variation. The aim of this study was to assess the biomarker potential of erythrocyte microRNAs for multiple sclerosis and assess the potential contribution of erythrocyte‐derived extracellular vesicle microRNAs to pathology.

**Methods:**

Erythrocytes were isolated from whole blood by density gradient centrifugation. Erythrocyte microRNAs of a discovery cohort (23 multiple sclerosis patients and 22 healthy controls) were sequenced. Increased expression of miR‐183 cluster microRNAs (hsa‐miR‐96‐5p, hsa‐miR‐182‐5p and hsa‐miR‐183‐5p) was validated in an independent cohort of 42 patients and 45 healthy and pathological (migraine) controls. Erythrocyte‐derived extracellular vesicles were created *ex vivo* and their microRNAs were sequenced. Targets of microRNAs were predicted using miRDIP.

**Results:**

Hsa‐miR‐182‐5p and hsa‐miR‐183‐5p were able to discriminate relapsing multiple sclerosis patients from migraine patients and/or healthy controls with 89‐94% accuracy and around 90% specificity. Hsa‐miR‐182‐5p and hsa‐miR‐183‐5p expression correlated with measures of physical disability and hsa‐miR‐96‐5p expression correlated with measures of cognitive disability in multiple sclerosis. Erythrocytes were found to selectively package microRNAs into extracellular vesicles and 34 microRNAs were found to be differentially packaged between healthy controls and multiple sclerosis patients. Several gene targets of differentially expressed and packaged erythrocyte microRNAs overlapped with multiple sclerosis susceptibility genes. Gene enrichment analysis indicated involvement in nervous system development and histone H3‐K27 demethylation.

**Conclusions:**

Erythrocyte miR‐183 cluster members may be developed into specific multiple sclerosis biomarkers that could assist with diagnosis and disability monitoring. Erythrocyte and their extracellular microRNAs were shown to target multiple sclerosis susceptibility genes and may be contributing to the pathophysiology via previously identified routes.

**Abbreviations**
ARCS: audio‐recorded cognitive screen
ARMSS: age‐related multiple sclerosis severity
AUC: area under the curve
BBB: blood‐brain barrier
cDNA: complementary DNA
CNS: central nervous system
CSF: cerebrospinal fluid
DMT: disease‐modifying therapy
DNA: deoxyribonucleic acid
EAE: experimental autoimmune encephalomyelitis
EDSS: expanded disability status scale
EV: extracellular vesicle
FDR: false discovery rate
GWAS: genome‐wide association study
HC: healthy control
HEPES: 4‐(2‐hydroxyethyl)‐1‐piperazineethanesulfonic acid
miRNA: microRNA
MP: migraine patient
MRI: magnetic resonance imaging
mRNA: messenger RNA
MS: multiple sclerosis
MSSS: multiple sclerosis severity score
NGS: next‐generation sequencing
NS: not significant
PBMC: peripheral blood mononuclear cell
PPMS: primary progressive multiple sclerosis
RISC: RNA‐induced silencing complex
RNA: ribonucleic acid
ROC: receiver operating characteristic
RRMS: relapsing‐remitting multiple sclerosis
RT‐qPCR: reverse‐transcription quantitative PCR
SD: standard deviation of the mean
sNfL: serum neurofilament light chain
SNP: single nucleotide polymorphism
SPMS: secondary progressive multiple sclerosis

## 1 INTRODUCTION

Multiple sclerosis is an autoimmune disease of the central nervous system (CNS). In multiple sclerosis, an assumed lymphocyte‐driven pathology causes the destruction of myelin and neurodegeneration, leading to the formation of sclerotic plaques.^1^ Multiple sclerosis affects approximately 2.5 million individuals worldwide,^2^ but incidence and prevalence are rising.^3^, ^4^

Genetic susceptibility interacting with environmental risk factors is thought to trigger the onset of multiple sclerosis, yet the exact cause and detailed pathological mechanisms remain to be elucidated.^1^, ^5^ The list of susceptibility genes linked to multiple sclerosis risk has been growing over the years and currently includes over 500 potential susceptibility genes and more than 200 genetic variants identified by several large genome‐wide association studies (GWAS).^6^ Not one of the genetic variants is necessary or sufficient for an individual to develop multiple sclerosis. Therefore, it is likely that several independent pathological factors lead to multiple sclerosis via different mechanisms.

Multiple sclerosis comprises three clinical phenotypes: relapsing‐remitting multiple sclerosis (RRMS), secondary progressive multiple sclerosis (SPMS) and primary progressive multiple sclerosis (PPMS). The majority of patients (around 85%) are diagnosed with relapse onset multiple sclerosis. RRMS patients suffer from transient inflammation‐driven demyelination or relapses.^7^ Neurodegeneration accumulates due to and independently of relapses.^8^ While remyelination of affected areas is possible in early disease, the CNS’ capacity to repair demyelination becomes exhausted over time and residual relapse disability starts to ensue. This gradual worsening marks the conversion to the progressive disease stage. SPMS is considered to be predominantly neurodegenerative, though relapses can still occur.^7^

Key players in multiple sclerosis are peripheral and CNS‐resident immune cells. It is widely accepted that peripheral immune cells gain access to the CNS via a damaged blood‐brain barrier (BBB) and initiate the destruction of myelin by creating an inflammatory environment and activating microglia.^1^ Erythrocytes may contribute to multiple sclerosis pathology through impaired antioxidant capacity and altered haemorheological features that may facilitate BBB damage.^9^ Erythrocytes are anucleate blood cells from the myeloid lineage that are primarily known for their role in respiratory gas transport.^10^, ^11^ Beyond their role as oxygen and carbon dioxide carriers, erythrocytes are involved in reducing oxidative stress and clearing immune complexes.^9^, ^12^

Current multiple sclerosis diagnosis is based on the exclusion of differentials, such as migraines, neuromyelitis optica spectrum disorder, infections, malignancy and vasculitis.^13^ Further, multiple sclerosis diagnoses rely heavily on clinical evidence and neurologist expertise. Paraclinical tests that support such a diagnosis are magnetic resonance imaging (MRI) of the brain and spinal cord and lumbar punctures to identify oligoclonal bands in the cerebrospinal fluid (CSF).^14^ None of these tests are specific to multiple sclerosis. For instance, non‐specific white matter lesions on MRI scans are also evident in migraine patients^15^ and cerebral small vessel disease,^16^ both of which are differential diagnoses of multiple sclerosis and need to be excluded for an accurate diagnosis. This highlights the need for more specific multiple sclerosis biomarkers to facilitate diagnostic decision making. To date, there are no blood‐borne biomarkers that are routinely used in clinical settings. Serum neurofilament light chain (sNfL) shows promise to be developed into a disease activity marker. sNfL is typically found in the cytoplasm of neurons, but is released into the CSF in response to neuronal damage. Following release into the CSF, sNfL is detectable at low concentration in the serum of multiple sclerosis patients^17^ using a specialised detection platform (Simoa).^18^ While sNfL is a very sensitive biomarker for multiple sclerosis, it is not specific. Increases in sNfL can be observed in all instances of CNS damage. For example, increased sNfL levels could also be detected in frontotemporal dementia^19^ and stroke.^20^ Therefore, sNfL may be suitable to detect disease activity multiple sclerosis patients, who have already been diagnosed, but may not serve as a diagnostic test. There is still a need for specific biomarkers to support multiple sclerosis diagnoses.

MicroRNAs (miRNAs) are small non‐coding RNA transcripts that are involved in post‐transcriptional regulation of gene expression and may prove to be specific biomarkers. In presence of an RNA‐induced silencing complex, miRNAs can bind to the 3′ untranslated region of messenger RNA (mRNA) transcripts through complementary base pairing and thereby cause translational repression or transcript degradation.^21^ Some miRNAs are transcribed as clusters or families. These miRNA clusters may target several genes within the same pathways, enabling increased epigenetic regulation of these pathways.^22^ Peripheral miRNAs have been previously assessed in the context of multiple sclerosis. However, the findings of previous studies are inconsistent. Such discrepancies may be attributed to the study of mixed cell populations, such as peripheral blood mononuclear cells (PBMCs) or the use of different assays/methods to survey these miRNAs.^23^

While human erythrocytes are thought to be translationally inactive,^24^ they contain abundance of miRNAs.^25^, ^26^, ^27^ Erythrocyte miRNAs have been previously implicated in diseases such as sickle cell disease,^26^ malaria^28^ and high altitude exposure.^29^ Erythrocytes are abundant in the circulation and can be easily obtained as part of other routine blood tests, making them a suitable biomarker source. Further, erythrocytes are a more uniform cell population than many of the immune cells previously assessed for multiple sclerosis miRNA biomarkers.^23^ The lack of transcriptional activity within erythrocytes hints at a more stable source of miRNAs.^24^ Together, this highlights the possibility erythrocyte miRNAs have in reducing the variability in miRNA signatures seen in the literature thus far.

Erythrocyte miRNAs may be left over from erythropoiesis where they regulate maturation^30^ or they may be involved in intercellular communication through extracellular vesicles (EVs).^26^, ^31^ EVs are small membrane‐bound vesicles that are released by most cells in response to stress or activation. The cargo inside these vesicles comprises a number of biologically active molecules ranging from lipids and carbohydrates to proteins and nucleic acids. Once released into the extracellular space, EVs can travel to recipient cells and alter the recipient cells’ state or function through the delivery of their cargo. Recipient cells may take up EVs through endocytosis or membrane fusion. EVs may also interact with recipient cells through ligand‐receptor binding.^32^ Research in the malaria field has shown that erythrocyte miRNAs may travel to neighbouring recipient cells. EV‐delivered miRNAs may then elicit changes in gene expression in recipient cells that facilitated BBB disruption and allowed the parasite to gain access to the CNS.^33^ While cerebral malaria and multiple sclerosis are not directly linked, BBB disruption is characteristic of both. Hsa‐miRNA‐451a, which was transferred from erythrocytes to endothelial cells through EVs, mediated the loss of barrier integrity in cerebral malaria.^33^ The same miRNA, hsa‐miR‐451a, was found at increased levels in multiple sclerosis patients' plasma EVs.^34^ Increased levels of plasma‐borne EVs have been reported in multiple sclerosis and linked to disease activity.^35^

Next‐generation sequencing (NGS) represents an unbiased approach to obtain complete miRNA profiles for the identification of disease‐specific signatures. Erythrocyte miRNA sequencing has been successfully performed by several research groups.^26^, ^27^, ^36^ We have previously reported differential miRNA expression in the erythrocytes of RRMS patients on various disease‐modifying therapies (DMTs).^37^ The objective of this study was to assess erythrocyte miRNAs in untreated multiple sclerosis patients and to determine their biomarker potential. Additionally, packaging of these miRNAs into EVs was assessed to explore the potential function of erythrocyte miRNAs as intercellular messengers carried by EVs. This is the first study to investigate erythrocyte‐derived EV miRNAs in multiple sclerosis.

## 2 MATERIALS AND METHODS

In this case‐control study, erythrocyte miRNA expression was determined by NGS and validated with a targeted approach and in an independent validation cohort. Diagnostic biomarker potential (multiple sclerosis vs controls) of candidate miRNAs was determined through receiver operating characteristic (ROC) curves. Correlation between erythrocyte miRNA expression and disability measures was assessed to determine the disability biomarker potential. Finally, erythrocyte‐derived EV miRNA levels were measured to identify whether disease‐specific packaging was evident and targets of differentially expressed and packaged miRNAs were predicted to determine potential involvement in multiple sclerosis pathogenesis.

### 2.1 Sample collection

#### 2.1.1 Ethics statement

Ethical approval was obtained from the University of Newcastle (H‐505‐0607) and the Hunter New England Health Ethics Committee (2019/ETH12346). Participants were recruited through the multiple sclerosis clinic at the John Hunter Hospital, the Hunter Medical Research Institute's volunteer register and Dr Andre Loiselle's migraine clinic (NSW, Australia). In accordance with the Declaration of Helsinki, all participants gave written informed consent prior to enrolment.

#### 2.1.2 Inclusion criteria and cohorts

Inclusion criteria for multiple sclerosis patients (cases) were that they had to (a) be ≥18 years old, (b) meet 2017 McDonald criteria for multiple sclerosis,^14^ (c) be treatmentnaïve or off DMT for >6 months prior to sample collection and (d) not have received corticosteroids in the 90 days prior to sample collection. Patients were excluded if they had an autoimmune condition other than multiple sclerosis. Healthy controls (HC) had to be (a) age‐matched to patients (±2 years) and (b) free of any autoimmune or (c) CNS disease. Migraines are part of the multiple sclerosis differential, therefore migraine patients were recruited as pathological controls. Migraine patients had to be (a) free of autoimmune disease and (b) CNS disease other than migraines. All participants who were pregnant or breastfeeding were excluded. To minimise potential confounders, only females were recruited.

We collected three independent cohorts for this study.

##### 2.1.2.1 Discovery cohort

Sample size was based on power calculations in G*Power 3.1.9. To obtain 90% power at *α* = .05, 12 samples per group (12 RRMS, 12 SPMS and 24 matched controls) were required. Eleven RRMS patients (stable), 12 SPMS patients and 22 age‐matched HCs were recruited. Patients were considered stable if they had not presented with any new clinical symptoms or gadolinium‐enhancing lesions on MRI scans in the 120 days pre‐collection and 90 days post‐collection.

##### 2.1.2.2 Validation cohort

Eighteen RRMS patients (stable), 14 relapsing patients, 13 SPMS patients, 27 matched HCs and 20 migraine patients were recruited. Active relapse was defined as new multiple sclerosis–related symptoms or evidence of gadolinium‐enhancing lesion(s) on MRI scans within one month of sample collection. Only patients that had not received corticosteroids prior to sample collection were included as relapsing patients.

##### 2.1.2.3 Erythrocyte‐derived EV cohort

Twelve stable RRMS patients, seven relapsing patients, eight SPMS patients and 16 matched HCs were recruited. Three of the stable RRMS patients, three of the relapsing patients, three of the SPMS patients and eight of the HCs were also part of the validation cohort.

Ten millilitres of whole blood were collected into lithium heparin‐coated vacutainers (Vacuette®, Greiner Bio‐One, Kremsmuenster, Austria) via venepuncture. Additional patient information was obtained through MSBase.^38^ Age‐related multiple sclerosis severity (ARMSS) scores and multiple sclerosis severity scores (MSSS) were calculated using the ms.sev package (v. 1.0.4) in R (v. 3.6.1). Patient demographics are listed in Table 1.

**TABLE 1:** Participant demographics

	RRMS (stable)	Relapse	HC (RRMS)	SPMS	HC (SPMS)	Migraine patients
**Discovery cohort**						
*N*	11	n/a	11	12	11	n/a
Age (years)	49.8 (±12.5)		48.4 (±13.3)	59.5 (±6.7)	61.2 (±7.0)	
Disease duration (years)	15.7 (±7.7)		n/a	23.0 (±11.6)	n/a	
Progression duration (years)	n/a			7.1 (±6.5)		
EDSS score	2.2 (±1.5)			6.3 (±0.9)		
Age at onset (years)	34.0 (±12.4)			36.5 (±10.1)		
Number of relapses	4 (±2)			5 (±3)		
Days since last relapse	1753 (±1825)			3005 (±2545)		
Most recent ARCS score^a^	98.3 (±16.0)			77.3 (±36.3)		
**Validation cohort**						
*N*	18	14	17	10	8	20
Age (years)	48.0 (±13.9)	37.6 (±9.7)	48.3 (±14.5)	64.0 (±13.9)	64.1 (±11.1)	44.4 (±12.6)
Disease duration (years)	18.3 (±13.9)	4.8 (±8.0)	n/a	27.2 (±12.0)	n/a	n/a
Progression duration (years)	n/a	n/a		3.2 (±2.6)		
EDSS score	1.6 (±1.4)	3.7 (±1.8)		5.7 (±1.7)		
Age at onset (years)	29.8 (±10.4)	32.6 (±8.7)		37.2 (±13.8)		
Number of relapses	4 (±3)	4 (±5)		3 (±2)		
Steroids ≤ 3 months (% yes)	0	28.6		0		
Days since last relapse	2491 (±4527)	13 (±15)		4843 (±2782)		
Most recent ARCS score^a^	90.8 (±10.0)	77.3 (±18.9)		93.8 (±15.9)		
**Erythrocyte‐derived extracellular vesicle cohort**						
*N*	12	7	11	8	5	n/a
Age (years)	43.4 (±10.5)	30.2 (±5.9)	43.1 (±11.2)	62.7 (±9.4)	56.4 (±8.0)	
Disease duration (years)	6.8 (±6.5)	4.8 (±3.8)	n/a	26.9 (±14.2)	n/a	
Progression duration (years)	n/a	n/a		13.0 (±17.4)		
EDSS score	2.1 (±1.3)	2.7 (±1.5)		5.7 (±2.2)		
Age at onset (years)	36.6 (±10.8)	25.3 (±5.4)		29.1 (±15.5)		
Number of relapses	4 (±4)	4 (±4)		5 (±4)		
Steroids ≤ 3 months (% yes)	0	42.9		0		
Days since last relapse	983 (±1178)	9 (±14)		5052 (±5759)		

*Note*. Data shown as mean (±SD).

Abbreviations: ARCS, audio‐recorded cognitive screen; EDSS, expanded disability status scale; HC, healthy control; n/a, not applicable; RRMS, relapsing‐remitting multiple sclerosis; SD, standard deviation of the mean; SPMS, secondary progressive multiple sclerosis.

a ARCS scores are only reported for patients who performed an ARCS within 12 months of sample collection (n = 31).

### 2.2 Blood processing

Erythrocytes were obtained via differential centrifugation with density gradient media (Lymphoprep, STEMCELL Technologies, Vancouver, Canada) as previously described.^37^ Erythrocyte purity was determined by flow cytometry (FITC‐conjugated anti‐CD235a antibody, Clone 2B7, BD Pharmingen, Franklin Lakes NJ, USA) as previously described.^37^ All samples met a purity cut‐off of 95%. Erythrocyte pellets (300 µL aliquots) were then frozen at −80°C until RNA was extracted in batches of 12.

We attempted to isolate erythrocyte‐derived EVs from plasma; however, we were unable to obtain sufficient EVs of acceptable purity for sequencing. Further, libraries constructed from plasma EVs did not pass quality control. Therefore, erythrocyte‐derived EVs were created *ex vivo* from purified erythrocytes. For erythrocyte‐derived EVs, erythrocytes were purified as described above from the EV cohort. Five millilitres of purified erythrocytes were then incubated with 4‐(2‐hydroxyethyl)‐1‐piperazineethanesulfonic acid (HEPES)‐buffered RPMI (Roswell Park Memorial Institute) (Thermo Fisher Scientific, Waltham MA, USA) media for 24 hours in 37°C and 5% CO_2_. Supernatants were harvested via a series of centrifugation steps (1500 × *g* for 10 minutes with break off, followed by 3000 × *g* for 15 minutes with break twice) and frozen at −80°C until RNA was extracted in batches of 10.

### 2.3 RNA extraction

#### 2.3.1 Erythrocytes

RNA was extracted from erythrocyte pellets with miRNeasy Mini kits (QIAGEN, Germany) as per manufacturer's protocol. Erythrocytes were lysed and homogenised by vortexing samples with QIAzol lysis reagent (QIAGEN, Hilden, Germany) for 1 minute. RNA concentration was determined with the Qubit 2.0 fluorometer (Thermo Fisher Scientific, Waltham MA, USA), using the broad‐range RNA assay (Invitrogen, Carlsbad CA, USA), and purity was checked on the Epoch Two microplate spectrophotometer (Millennium Science, Mulgrave VIC, Australia).

#### 2.3.2 Erythrocyte‐derived EVs

RNA was extracted from 4 mL of erythrocyte supernatant using ExoRNeasy Serum/Plasma Maxi kits (QIAGEN, Hilden, Germany), which isolate EVs as part of the RNA extraction process. RNA concentration was determined with the Qubit 2.0 fluorometer, using the high‐sensitivity RNA assay (Invitrogen, Carlsbad CA, USA). Due to low yields, purity was not checked. A negative control (cell culture medium only) was also prepared.

RNA samples were frozen at −80°C until NGS library preparation or complementary DNA (cDNA) synthesis for reverse‐transcription quantitative PCR (RT‐qPCR).

### 2.4 Next‐generation sequencing

#### 2.4.1 Erythrocyte RNA

RNA from the discovery cohort was subjected to NGS on a MiSeq (Illumina, San Diego CA, USA) platform. Small RNA library preparation was performed using the TruSeq small RNA library preparation kits (Illumina, San Diego CA, USA). Libraries were pooled and run on an agarose gel for size exclusion prior to being purified with the Wizard SV Gel and PCR Clean‐Up system (Promega, Madison WI, USA). Quality control of NGS libraries was performed on the Tape Station (Agilent Technologies, Santa Clara CA, USA). Libraries were sequenced on four different flow cells for 37 cycles (MiSeq reagent kit v2, 50 cycles; Illumina, San Diego CA, USA) in a single‐end fashion, aiming for one million reads per sample.

#### 2.4.2 Erythrocyte‐derived EV RNA

RNA from the EV cohort and the negative control (RNA extraction from culture medium) were subjected to library preparation. MicroRNA libraries were created with the QIAseq miRNA library kit (QIAGEN, Hilden, Germany). This kit was chosen over the Illumina library preparation kit, as its input requirement was lower and could accommodate the low RNA yields from erythrocyte‐derived EVs. Libraries were pooled and sequenced in single‐end fashion on a NextSeq 500 flow cell (NextSeq 500/550 High Output v2.5 kit , 150 cycles; Illumina, San Diego CA, USA).

### 2.5 Reverse‐transcription quantitative PCR

Samples from the discovery and the validation cohort (including samples that did not pass quality control in the discovery cohort) were subjected to cDNA synthesis for RT‐qPCR. The commercially available TaqMan Advanced cDNA synthesis kit (Thermo Fischer Scientific, Waltham MA, USA) was used for cDNA synthesis. RNA input was 10 ng at 5 ng/µL. Quantitative PCR assays were performed using TaqMan Advanced miRNA probes (Thermo Fischer Scientific, Waltham MA, USA). The following probes were used: hsa‐miR‐96‐5p (478215_mir), hsa‐miR‐18a‐5p (478551_mir), hsa‐let‐7i‐5p (478375_mir), hsa‐miR‐183‐5p (477937_mir), hsa‐miR‐30a‐5p (479448_mir), hsa‐miR‐106b‐3p (477866_mir), hsa‐miR‐191‐5p (477952_mir), hsa‐miR‐103a‐3p (478253_mir), hsa‐miR‐629‐5p (478183_mir), hsa‐miR‐30e‐3p (478388_mir), hsa‐miR‐1307‐3p (483036_mir), hsa‐miR‐1246 (477881_mir) and hsa‐miR‐182‐5p (477935_mir). Hsa‐miR‐152‐3p (477921_mir) was used as an endogenous control.^26^, ^37^ All qPCR plates included no template controls, RNA controls and no reverse‐transcriptase controls. Relative expression was calculated using the 2^−(^ *^Ct^* ^miRNA of interest –^ *^Ct^* ^hsa‐miR‐152‐3p)^ method.

### 2.6 Target prediction and gene enrichment analysis

Targets of differentially expressed and packaged miRNAs were predicted using miRDIP 4.1, an integrative online database.^39^ Only targets that met the score class ‘Very High (Top 1%)’ were included. Gene enrichment analysis of targets was performed using GO Enrichment Analysis.^40^ Multiple sclerosis susceptibility genes were taken from the International Multiple Sclerosis Genetics Consortium GWAS (Table S19).^6^ Duplicate susceptibility genes were removed prior to analysis.

### 2.7 Statistical analysis

#### 2.7.1 Sequencing data

Data was analysed as described by Cordero et al.^41^ Briefly, adapters were trimmed with cutadapt. Sequencing reads were aligned against miRNAs deposited in miRBase (v. 22) with SHiMPS aligner and differential expression was computed in DESeq2. QIAGEN unique molecular identifiers were not used to allow erythrocyte and erythrocyte‐derived EV miRNA counts to be analysed together. Samples that did not pass quality control due to low reads were excluded prior to analysis. MicroRNAs with less than 10 reads (average) per sample were excluded prior to running DESeq2 (run in R v. 3.6.1). MicroRNAs with fold changes of ≥2 or ≤0.5 were considered differentially expressed between groups. Log fold change shrinkage was performed using the ‘apeglm’ package within DESeq2.

#### 2.7.2 RT‐qPCR

Statistical analysis was performed in R v. 3.6.1 and differential expression was determined through Kolmogorov‐Smirnov tests. A *P*‐value of less than .05 was deemed statistically significant.

#### 2.7.3 Biomarker analysis

ROC curve analysis was carried out in R v. 3.6.1. Accuracy was estimated using area under the curve (AUC) and thresholds that maximised both sensitivity and specificity were chosen; 95% CI intervals were reported. Correlations between the expression of different miRNAs were determined using Pearson's correlation coefficients, and correlations between miRNA expression and other clinical parameters were determined using Spearman's rho based on distribution of the data and the relationship between the variables. *P*‐values were corrected for multiple testing using the Benjamini‐Hochberg method.

## 3 RESULTS

As erythrocyte miRNAs are not well characterised and to eliminate potential confounders associated with sex in the small cohorts of this study, only female participants were recruited and controls were age‐matched (±2 years). Patient demographics are summarised in Table 1. Erythrocyte purity was determined by flow cytometry as previously described.^37^ Mean erythrocyte purity across all samples was 96.08%. The average RNA yield per 300 µL erythrocyte pellet was 1589.4 ng (±826.1 ng).

### 3.1 Next‐generation sequencing of erythrocyte microRNAs (discovery cohort)

The discovery cohort was used for NGS. NGS runs yielded between 12 170 404 and 13 459 251 reads with more than 95% of base calling scores ≥Q30 across all runs. More than 97% of reads could be aligned to mature miRNAs deposited in miRBase (v. 22) across all four runs. Two RRMS samples and one SPMS sample were removed from the discovery cohort prior to analysis due to low reads. Their matched HCs were also removed.

NGS detected differential erythrocyte miRNA expression in multiple sclerosis patients compared to HCs (Supporting Information File 1). Ten miRNAs were differentially expressed in multiple sclerosis patient erythrocytes (RRMS and SPMS combined, n = 20) compared to HCs (n = 18). Four of these showed decreased expression in multiple sclerosis. Of the differentially expressed miRNAs, hsa‐miR‐183‐5p, hsa‐miR‐30a‐5p, hsa‐miR‐191‐5p, hsa‐miR‐18a‐5p, hsa‐let‐7i‐5p, hsa‐miR‐106b‐3p and hsa‐miR‐1307‐3p were chosen for validation based on significance.

Looking at multiple sclerosis disease courses individually, five erythrocyte miRNAs showed decreased and seven increased expression in stable RRMS (n = 9) compared to HCs (n = 9). As expected, many of these overlapped with differentially expressed miRNAs identified in the multiple sclerosis versus HC comparison. In addition to the miRNAs listed above, hsa‐miR‐629‐5p, hsa‐miR‐30e‐3p, hsa‐miR‐103a‐3p and hsa‐miR‐1246 were also chosen for validation. Additionally, hsa‐miR‐96‐5p, which was differentially expressed in RRMS patients compared to HCs, was also included in the targeted assay as it was identified in the discovery phase of our previous erythrocyte miRNA study.^37^

Hsa‐miR‐183‐5p showed decreased expression in SPMS (n = 11) compared to HCs (n = 9) and hsa‐miR‐30a‐5p showed decreased expression in SPMS compared to RRMS in a direct comparison.

### 3.2 Reverse‐transcription qPCR of erythrocyte microRNAs (discovery cohort)

Reverse‐transcription qPCR is a less sensitive method than NGS and significance threshold was not reached for any candidate miRNA using this method. However, differential expression trends were confirmed for hsa‐miR‐18a‐5p, hsa‐miR‐96‐5p and hsa‐miR‐183‐5p. Hsa‐miR‐1246 could not be detected by RT‐qPCR and was not included in further analyses (PCR efficiencies are shown in Supporting Information File 2).

### 3.3 Validation study of erythrocyte microRNAs (independent validation cohort)

To further validate NGS results, expression of hsa‐miR‐18a‐5p, hsa‐miR‐96‐5p and hsa‐miR‐183‐5p was assessed in an independent validation cohort. Hsa‐miR‐183‐5p and hsa‐miR‐96‐5p are part of the three‐member miR‐183 cluster.^22^ Because miRNA clusters tend to be transcribed together, it was reasoned that the third cluster member, hsa‐miR‐182‐5p, may also show altered expression. Therefore, hsa‐miR‐182‐5p was included in the validation study. Samples that were not included in the discovery cohort analysis due to low‐sequencing reads were included in the validation cohort.

**FIGURE 1:**
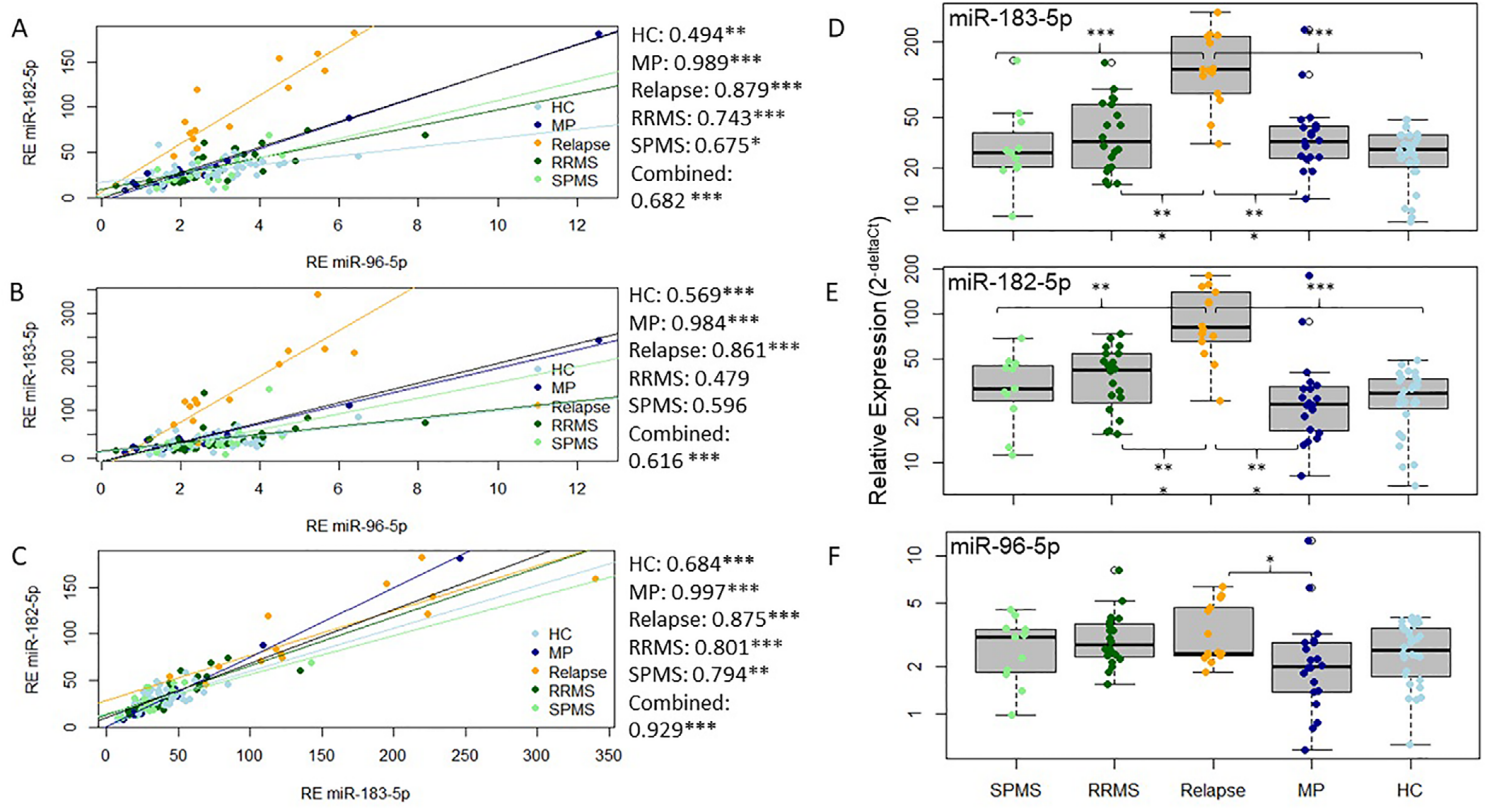
Relative expression (2^−deltaCt^) of miR‐183 cluster members in erythrocytes. A‐C, Discovery and validation cohort. Correlation between the expression of (A) hsa‐miR‐96‐5p and hsa‐miR‐182‐5p, (B) hsa‐miR‐96‐5p and hsa‐miR‐183‐5p and (C) hsa‐miR‐183‐5p and hsa‐miR‐182‐5p in the different groups (HC: light blue, n = 45; MP: dark blue, n = 20; relapse: orange, n = 14; RRMS: dark green, n = 27; SPMS: light green, n = 21) and overall (black line). Pearson's correlation coefficients are shown on the side. D‐F, Validation cohort only. Relative expression of (D) hsa‐miR‐183‐5p, (E) hsa‐miR‐182‐5p and (F) hsa‐miR‐96‐5p is shown in SPMS patients (n = 10, SPMS, light green), stable RRMS patients (n = 18, RRMS, dark green), relapsing patients (n = 14, relapse, orange), migraine patients (n = 20, MP, dark blue) and HCs (n = 25, HC, light blue). White circles represent outliers defined as deviating ≥1.5‐fold from the upper/lower quartile. HC, healthy control; MP, migraine patient; RE, relative expression; RRMS, relapsing‐remitting multiple sclerosis; SPMS, secondary progressive multiple sclerosis; ***P < .001; ** *P* < .01; *P < .05

MiR‐183 cluster members were expressed in coordinated fashion. Relative expression of all three miR‐183 cluster members correlated with each other in the different groups (HCs, migraine patients, stable RRMS patients, relapsing patients and SPMS patients), and overall in the combined discovery and validation cohort (Figure 1A‐C). The correlation appeared strongest between hsa‐miR‐182‐5p and hsa‐miR‐183‐5p, which were found to be expressed at similar levels in erythrocytes. Expression of hsa‐miR‐96‐5p was much lower in all the groups (Figure 1).

MiR‐183 cluster members showed increased expression during relapse. In relapsing patients, both hsa‐miR‐183‐5p and hsa‐miR‐182‐5p showed increased expression (*P* < .001) compared to stable RRMS patients (hsa‐miR‐183‐5p: 3.15‐fold; hsa‐miR‐182‐5p: 2.44‐fold), SPMS patients (hsa‐miR‐183‐5p: 3.77‐fold; hsa‐miR‐182‐5p: 2.80‐fold), HCs (hsa‐miR‐183‐5p: 5.22‐fold; hsa‐miR‐182‐5p: 3.46‐fold) and migraine patients (hsa‐miR‐183‐5p: 3.15‐fold; hsa‐miR‐182‐5p: 2.83‐fold). Additionally, multiple sclerosis patients’ (stable RRMS and SPMS combined, n = 28) erythrocytes harboured increased levels of hsa‐miR‐182‐5p (1.36‐fold, *P* < .01) compared to HCs (*n* = 25). Stable RRMS patients had higher expression of hsa‐miR‐182‐5p compared to HCs (1.44‐fold, *P* < .01). While hsa‐miR‐96‐5p displayed trends of increased expression during relapse compared to all other groups, this trend was only significant when compared to migraine patients (1.29‐fold, *P* < .05) (Figure 1D‐F).

Hsa‐miR‐18a‐5p was not found to be differentially expressed in the validation cohort (data not shown).

### 3.4 Biomarker potential and correlations with clinical data

Biomarker potential and correlations between miRNA‐183 cluster members and other clinical measures were assessed using the combined relative expression data (RT‐qPCR) from the discovery and validation cohort.

**FIGURE 2:**
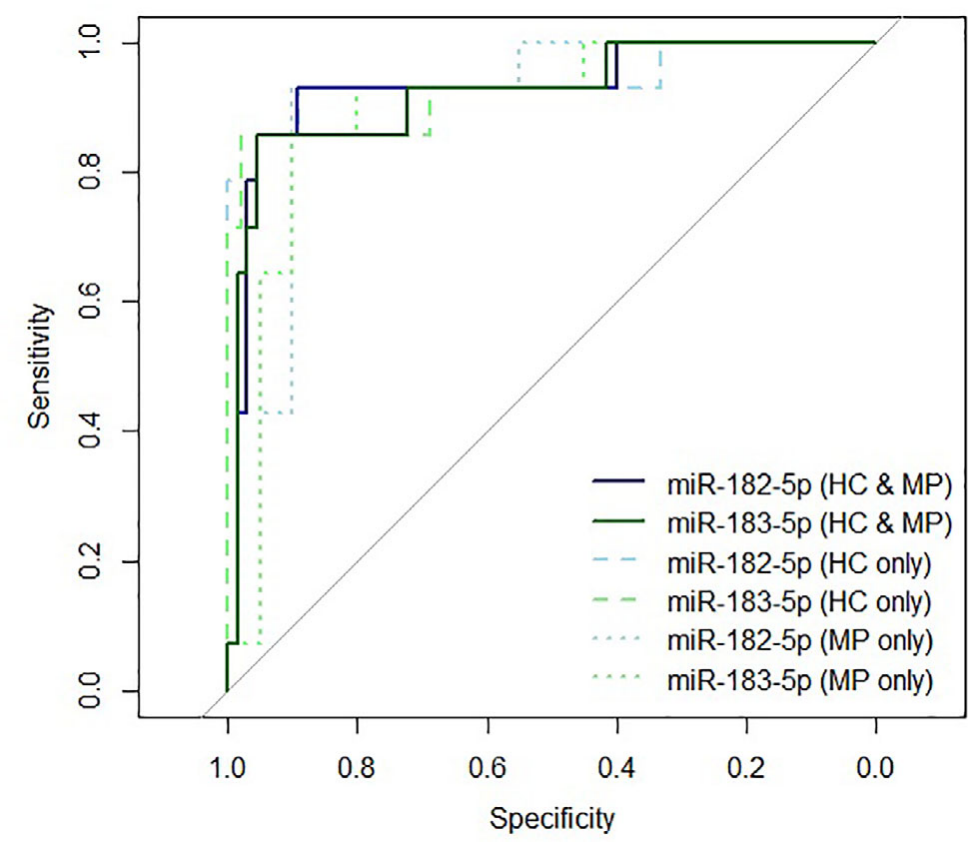
Receiver operating characteristics curves for hsa‐miR‐182‐5p and hsa‐miR‐183‐5p. Receiver operating characteristic curves show sensitivity and specificity (reverse axis) in distinguishing relapsing multiple sclerosis patients (n = 14) from all controls (HC and MP; n = 65; solid line), from HCs only (n = 45; dashed line) and from migraine patients only (n = 20; dotted line). Curves associated with hsa‐miR‐182‐5p are shown in blue and light blue, and curves associated with hsa‐miR‐183‐5p are shown in green and light green. HC, healthy control; MP, migraine patient

Hsa‐miR‐182‐5p and hsa‐miR‐183‐5p showed biomarker potential for relapsing multiple sclerosis patients. To determine the biomarker potential of erythrocyte‐derived miR‐183 cluster expression, ROC curve analysis was carried out. Both hsa‐miR‐182‐5p and hsa‐miR‐183‐5p showed potential to distinguish relapsing multiple sclerosis patients (n = 14) from the combined control groups (HCs and migraine patients, n = 65), as well as HCs (n = 45) and migraine patients (n = 20) independently. Accuracy was estimated using AUC and lay between 89.6% and 94.3% for the different comparators. Further, when choosing threshold values that allowed for optimal sensitivity and specificity, both miRNAs could exclude migraine patients with 90% specificity (Figure 2, Table 2). Combining the two miRNAs in a logistic regression model did not improve sensitivity, specificity or accuracy (data not shown).

**TABLE 2:** Receiver operating characteristic curve values

	hsa‐miR‐182‐5p vs			hsa‐miR‐183‐5p vs		
Relapse	All controls	HC	MP	All controls	HC	MP
AUC	0.930	0.943	0.900	0.921	0.932	0.896
AUC 95% CI	0.842‐1	0.849‐1	0.784‐1	0.831‐1	0.839‐1	0.779‐1
Threshold^a^	43.226	51.972	43.078	68.275	68.275	59.417
Sensitivity	0.929	0.857	0.929	0.857	0.857	0.857
Specificity	0.892	0.978	0.900	0.954	0.978	0.900

Abbreviations: AUC, area under the curve; CI, confidence interval; HC, healthy control; MP, migraine patient.

a Thresholds that maximised both sensitivity and specificity are shown.

**FIGURE 3:**
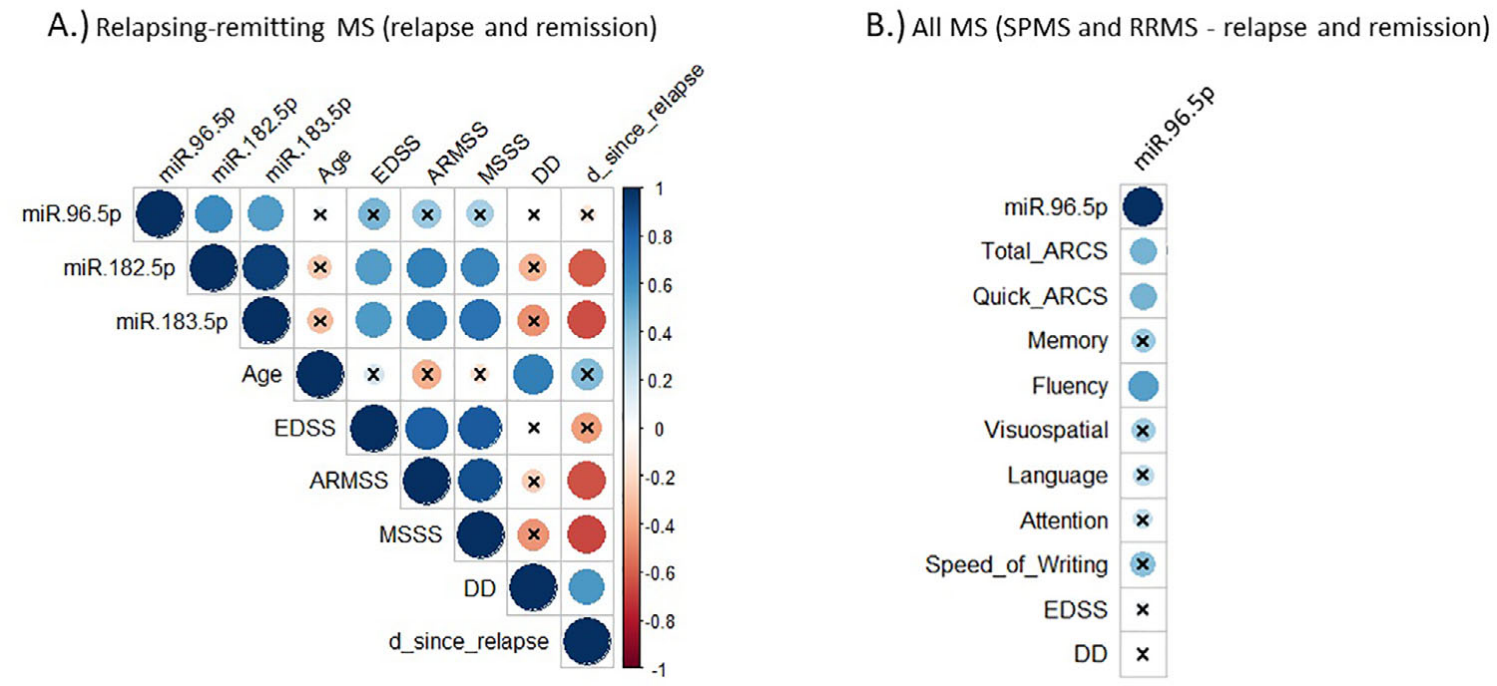
Spearman's rho correlation matrix of miR‐183 cluster members and clinical measures. A, Correlation matrix of miR‐183 cluster member expression, age, EDSS scores, ARMSS scores, MSSS, disease duration and days since last relapse for all RRMS patients (stable and relapsing, n = 41). B, Correlation between hsa‐miR‐96‐5p expression and ARCS, its subdomain scores (memory, fluency, visuospatial, language, attention, speed of writing), EDSS scores and disease duration for all multiple sclerosis patients who have undertaken an ARCS within 12 months of sample collection (n = 31). Positive correlations are shown in blue and negative correlations in red. Nonsignificant correlations are crossed out (*P*‐values adjusted using Benjamini‐Hochberg method; *α* =.05). Quick ARCS scores are ARCS scores that have not been adjusted for education and age. ARCS, audio‐recorded cognitive screen; ARMSS, age‐related multiple sclerosis severity; d_since_relapse, days since last relapse; DD, disease duration; EDSS, expanded disability status scale; MS, multiple sclerosis; MSSS, multiple sclerosis severity score; RRMS, relapsing‐remitting multiple sclerosis; SPMS, secondary progressive multiple sclerosis

MicroRNA‐183 cluster members correlated with measures of physical and cognitive disability in multiple sclerosis (Figure 3, Table 3). In all RRMS patients (stable and relapsing, n = 41), both hsa‐miR‐182‐5p and hsa‐miR‐183‐5p expression were positively correlated with expanded disability status scale (EDSS) scores, ARMSS scores and MSSS. Furthermore, expression of hsa‐miR‐182‐5p and hsa‐miR‐183‐5p decreased with increasing time since patients’ last relapse and showed trends of decreasing expression with increasing disease duration. However, the latter may be related to a reduction in recent relapses in patients with longer disease duration (Figure 3A, Table 3). Beyond the domain of physical disability reflected by EDSS and related scores, multiple sclerosis patients suffer from cognitive disability. One way of assessing cognitive disability are cognitive screening tests, such as the audio‐recorded cognitive screen (ARCS).^42^ In all multiple sclerosis patients [SPMS and RRMS (stable and relapsing)] who had performed an ARCS within 12 months of sample collection (n = 31), hsa‐miR‐96‐5p was positively correlated with patients’ total ARCS score, as well as fluency, one of the ARCS’ subdomains. Trends of increasing expression with increasing score in the other subdomains (memory, visuospatial, language, attention and speed of writing) were also observed (Figure 3B, Table 3).

**TABLE 3:** Spearman's rho of correlations with clinical parameters

	hsa‐miR‐182‐5p		hsa‐miR‐183‐5p	
	Spearman's rho	Adj. *P*‐value	Spearman's rho	Adj. *P*‐value
EDSS score^a^	0.552	.012	0.565	9.0E‐03
ARMSS score	0.676	2.0E‐04	0.701	4.6E‐05
MSSS	0.659	3.0E‐04	0.733	8.1E‐06
Days since relapse	−0.609	8.0E‐04	−0.642	2.1E‐04

		hsa‐miR‐96‐5p	
		Spearman's rho	Adj. *P*‐value
Total ARCS score^b^		0.465	.024
Quick ARCS score		0.466	.024
Subdomain scores	Memory	0.362	.117
	Fluency	0.548	.004
	Visuospatial	0.342	.143
	Language	0.261	.323
	Attention	0.243	.055
	Speed of writing	0.417	.011

a ^)^Data shown for RRMS patients (stable and relapse; n = 41).

b ^)^ Data shown for all multiple sclerosis patients whohave undertaken an ARCS within 12 months of sample collection (n = 31).

Abbreviation: ARMSS, age‐related multiple sclerosis severity; ARCS, audio‐recorded cognitive screen. EDSS, expanded disability status scale; MSSS, multiple sclerosis severity score; RRMS, relapsing‐remitting multiple sclerosis.

### 3.5 Erythrocyte‐derived EV microRNAs

Human erythrocytes are translationally inactive.^24^ Therefore, the role of miRNAs within them remains to be elucidated. One hypothesis is that erythrocyte miRNAs may be packaged into EVs and travel to translationally active recipient cells to carry out their function.^26^, ^33^ As there was an increase in erythrocyte‐derived EVs in the plasma of relapsing multiple sclerosis patients (Supporting Information File 3), we wanted to determine whether erythrocytes may be packaging their miRNAs into EVs in a disease‐specific manner in multiple sclerosis. Therefore, erythrocyte‐derived EVs were collected from the supernatant of incubated erythrocytes. Total RNA was extracted and small RNA libraries sequenced.

Sequencing of erythrocyte‐derived EV miRNAs yielded between three and nine million reads per sample with 96.01% of base calling scores ≥Q30. Around 20% of all sample reads were mappable to miRNAs deposited in miRBase (v. 22.0). Only 0.6% of the negative control reads were mappable.

**FIGURE 4:**
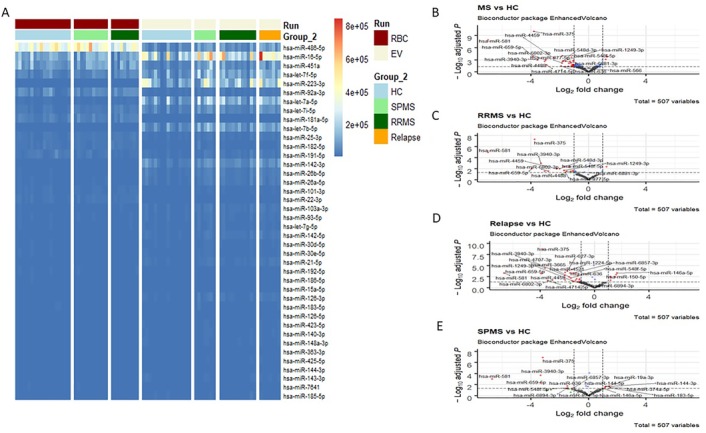
Erythrocyte and erythrocyte‐derived extracellular vesicle microRNA expression. A, Heat map of the 40 most abundant erythrocyte miRNAs in erythrocytes (RBC, dark red) and in erythrocyte‐derived EVs (EV, beige) by subject group (HC: light blue; relapse: orange; RRMS: dark green; SPMS: light green) based on sequencing data. B‐E, Volcano plots of erythrocyte‐derived EV miRNA sequencing results. B, All multiple sclerosis patients (RRMS and SPMS combined, *n* = 26) versus HCs (n = 16). C, RRMS (n = 12) versus HCs (n = 16). D, Relapsing patients (n = 7) versus HCs (n = 16). E, SPMS patients (n = 7) versus HCs (n = 16). Differentially enriched miRNAs are highlighted in red. Dotted lines represent cut‐off values: adjusted *P*‐value < .05 and fold change ≥2 or ≤0.5. EV, extracellular vesicle; HC, healthy control; miRNA, microRNA; MS, multiple sclerosis; RBC, red blood cell (erythrocyte); RRMS, relapsing‐remitting multiple sclerosis; SPMS, secondary progressive multiple sclerosis

Erythrocyte‐derived EV miRNAs were selectively packaged. The majority of highly expressed erythrocyte miRNAs were also highly enriched in erythrocyte‐derived EVs. However, hsa‐miR‐486‐5p (*P* = 4.58E‐125), hsa‐miR‐451a (*P* = 8.00E‐49) and hsa‐miR‐92‐3p (*P* = 6.25E‐76) showed decreased levels in EVs compared to erythrocytes, and hsa‐miR‐223‐5p (*P* ˂ 2.225074E‐308), hsa‐let‐7a‐5p (*P* = 2.89E‐42), hsa‐let‐7b‐5p (*P* = 5.15E‐154) and hsa‐miR‐142‐3p (*P* ˂ 2.225074E‐308) showed increased levels in EVs compared to erythrocytes (Figure 4A).

Erythrocyte‐derived EV miRNAs showed differential enrichment between multiple sclerosis patients and HCs. There were 34 differentially enriched erythrocyte‐derived EV miRNAs between multiple sclerosis patients (n = 26) and HCs (n = 16). Three of these miRNAs were increased in multiple sclerosis patients compared to HCs. Additionally, there were 17 differentially enriched erythrocyte‐derived EV miRNAs between stable RRMS patients and HCs, 15 between SPMS patients and HCs, and 36 between relapsing patients and HCs. Hsa‐miR‐148‐5p was increased in relapsing patients’ EVs compared to stable RRMS patients’ (fold change: 2.62, *P* = .003). No differences in EV miRNAs were observed between SPMS and stable RRMS patients or SPMS patients and relapsing patients (Figure 4B‐E, Supporting Information File 5). The miR‐183 cluster was not found to be differentially packaged into erythrocyte‐derived EVs in multiple sclerosis.

### 3.6 MicroRNA target prediction and gene enrichment analysis

**FIGURE 5:**
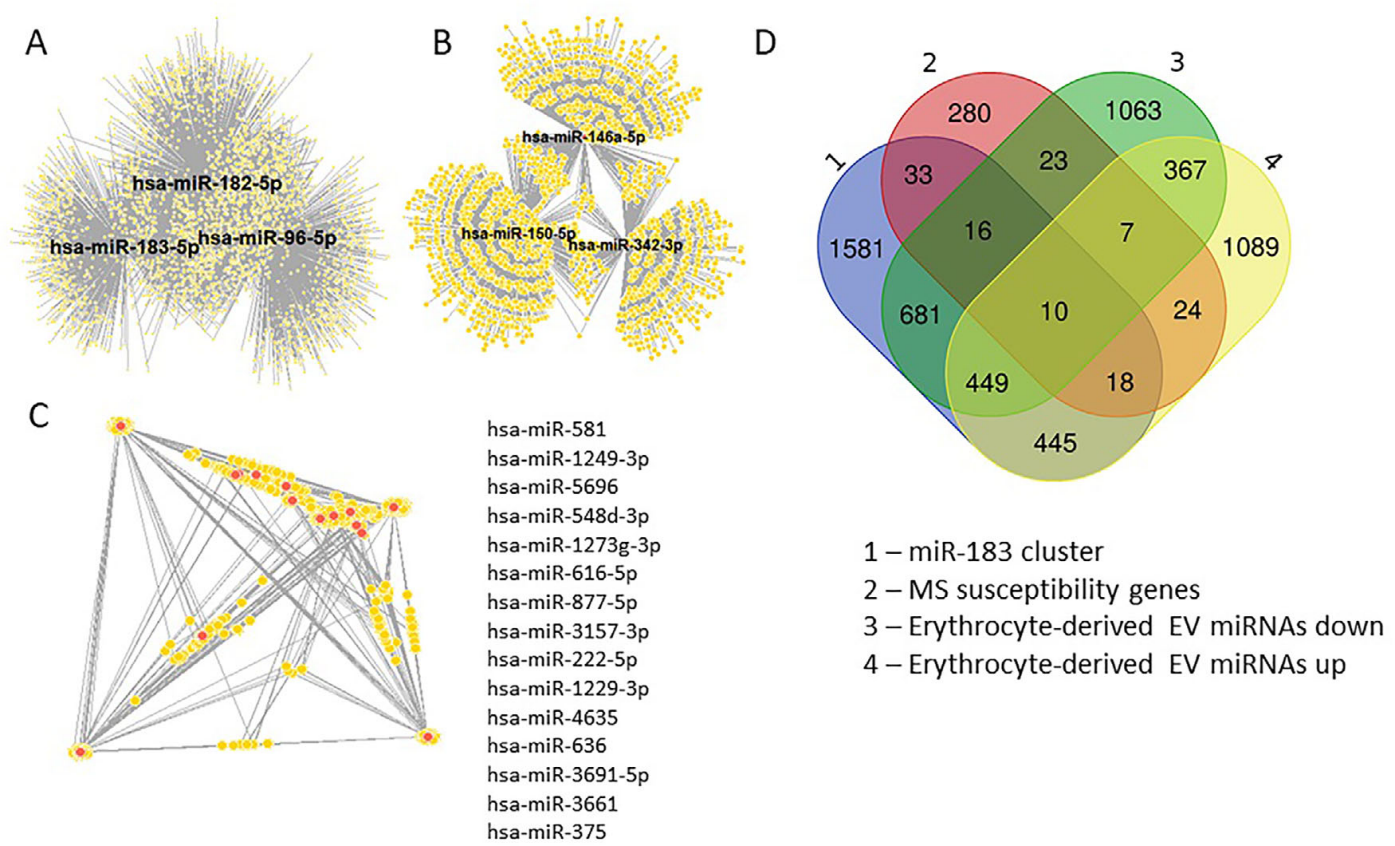
Predicted targets of differentially expressed and packaged (extracellular vesicle) erythrocyte microRNAs. A‐C, Predicted targets (yellow circles) of (A) the miR‐183 cluster, (B) miRNAs that were increased in multiple sclerosis erythrocyte‐derived EVs compared to HCs and (C) miRNAs that were decreased in multiple sclerosis erythrocyte‐derived EVs compared to HCs (red circles). D, Overlap between predicted miRNA targets for all three groups (A‐C) and multiple sclerosis susceptibility genes. Detailed information is provided in Supporting Information File 5. EV, extracellular vesicle; HC, healthy control; miRNA, microRNA; MS, multiple sclerosis

To assess the potential function of erythrocyte miRNAs and erythrocyte‐derived EV miRNAs in multiple sclerosis, targets of differentially expressed and packaged miRNAs (multiple sclerosis versus HCs) were predicted using an online database.^39^ Figure 5A‐C and Supporting Information File 5 show the targets shared by multiple miRNAs. Multiple miRNAs targeting the same transcript may amplify the effects of post‐transcriptional regulation.

Several of the miRNA gene targets have been previously flagged as multiple sclerosis susceptibility genes. Using the multiple sclerosis susceptibility gene list from the 2019 GWAS,^6^ miRNA targets that have already been linked to multiple sclerosis were identified. Of the predicted targets for the miR‐183 cluster and differentially enriched erythrocyte‐derived EV miRNAs, 131 overlapped with the proposed multiple sclerosis susceptibility genes (Figure 5D, Supporting Information File 5).

Additionally, gene enrichment analysis was carried out on the targets of differentially enriched erythrocyte‐derived EV miRNAs. MicroRNAs that were decreased in multiple sclerosis patients compared to HCs were enriched in several pathways linked to nervous system development and histone H3‐K27 demethylation (7.9‐fold enrichment, *P* = .041). MicroRNAs that were increased in multiple sclerosis patients erythrocyte‐derived EVs were enriched in pathways associated with cardiac muscle contraction (Table 4).

**TABLE 4:** Gene enrichment analysis of erythrocyte‐derived extracellular vesicle microRNA targets

Pathway	Fold enrichment	*P*‐value	FDR‐adj. *P*‐value
**Erythrocyte‐derived EV microRNAs (decreased in MS compared to HC)**			
Glossopharyngeal nerve development (GO:0021563)	7.9	2.77E‐03	4.13E‐02
Histone H3‐K27 demethylation (GO:0071557)	7.9	2.77E‐03	4.12E‐02
Noradrenergic neuron differentiation (GO:0003357)	6.77	1.71E‐03	2.81E‐02
Maintenance of postsynaptic specialization structure (GO:0098880)	5.92	2.70E‐03	4.04E‐02
Ventricular compact myocardium morphogenesis (GO:0003223)	5.92	2.70E‐03	4.04E‐02
Insulin‐like growth factor receptor signalling pathway (GO:0048009)	5.64	1.40E‐04	3.33E‐03
Commissural neuron axon guidance (GO:0071679)	5.26	9.12E‐04	1.64E‐02
Peripheral nervous system neuron development (GO:0048935)	5.26	9.12E‐04	1.64E‐02
Peripheral nervous system neuron differentiation (GO:0048934)	5.26	9.12E‐04	1.64E‐02
**Erythrocyte‐derived EV microRNAs (increased in MS compared to HC)**			
AV node cell to bundle of His cell communication (GO:0086067)	5.99	1.03E‐03	2.98E‐02
Membrane depolarization during cardiac muscle cell action potential (GO:0086012)	5.53	6.38E‐05	2.80E‐03
Neuromuscular process controlling posture (GO:0050884)	4.53	8.85E‐04	2.63E‐02
Negative regulation of smooth muscle cell differentiation (GO:0051151)	4.53	8.85E‐04	2.63E‐02
Hippo signalling (GO:0035329)	4.12	1.39E‐04	5.51E‐03
Regulation of smooth muscle cell differentiation (GO:0051150)	3.74	1.72E‐04	6.61E‐03
Membrane depolarization during action potential (GO:0086010)	3.71	3.13E‐04	1.12E‐02
Regulation of heart rate by cardiac conduction (GO:0086091)	3.6	8.61E‐05	3.62E‐03
Cell‐cell signalling involved in cardiac conduction (GO:0086019)	3.48	1.33E‐03	3.71E‐02
mRNA destabilization (GO:0061157)	3.42	9.28E‐04	2.74E‐02

Abbreviations: EV, extracellular vesicle; FDR‐adj., false discovery rate‐adjusted; HC, healthy control; MS, multiple sclerosis.

## 4 DISCUSSION

In this study, erythrocyte miRNAs and erythrocyte‐derived EV miRNAs were predicted to target genes previously implicated in multiple sclerosis susceptibility. This finding highlights that erythrocyte miRNAs are reflective of multiple sclerosis pathophysiology. Further, erythrocyte miRNAs show multiple sclerosis–specific biomarker potential that may assist in ruling out differential diagnoses and monitor disability.

### 4.1 Erythrocyte microRNAs as biomarkers

Multiple sclerosis diagnosis is currently based on the exclusion of other differentials, which often leaves significant uncertainty. Consequently, more objective biomarkers are needed to aid diagnostic decisions, which may lead to earlier treatment initiation.^43^ The importance of the latter in reducing the disability burden in multiple sclerosis has been shown by numerous studies.^43^, ^44^ While several attempts to develop leukocyte miRNA biomarkers for multiple sclerosis can be found in the literature, the results of these studies are highly inconsistent.^23^, ^45^ This may be a result of post‐collection induction of miRNAs in nucleated cells, the use of different assays or even heterogeneity of the disease. Erythrocytes may overcome one of these barriers to biomarker development. Being transcriptionally inactive,^24^ their miRNA profiles may be more stable. In fact, evidence for erythrocyte miRNA stability can be found in the literature.^46^ To determine biomarker specificity, a cohort of migraine patients was included in this study. Similar to multiple sclerosis patients, migraine patients may present with white matter lesions on MRI scans.^15^ As a result, migraines are part of the differential diagnoses that need to be considered in multiple sclerosis. Erythrocyte hsa‐miR‐182‐5p and hsa‐miR‐183‐5p showed increased expression during relapse compared to stable RRMS, SPMS, HCs and migraine patients (Figure 1). Differences in hsa‐miR‐182‐5p and hsa‐miR‐183‐5p expression were able to differentiate relapsing multiple sclerosis patients from HCs and migraine patients with 92.1% and 93.0% accuracy, respectively, and 89.2% and 95.4% specificity, respectively (Figure 2, Table 2). Additionally, hsa‐miR‐183‐5p and hsa‐miR‐182‐5p expression correlated with EDSS scores and related measures of physical disability. Hsa‐miR‐96‐5p, the third member of the miR‐183 cluster, correlated with cognitive disability (determined by ARCS; Figure 3, Table 3). This strongly indicates the diagnostic and disability biomarker potential of these miRNAs for multiple sclerosis, which may be used in combination with current MRI biomarkers. Increased miR‐183 cluster expression has been observed in systemic lupus erythematosus murine model lymphocytes,^47^ in osteoblasts of rheumatoid arthritis patients^48^ and multiple sclerosis murine model Th17‐lymphocytes.^49^ With erythrocyte miRNA investigations still in their infancy, future studies should aim to investigate the expression of miR‐183 cluster miRNAs in the erythrocytes of patients with other autoimmune diseases.

Although the miR‐183 cluster is transcribed as one primary miRNA,^22^ there appeared to be differences in expression between hsa‐miR‐96‐5p and the other two members of the miR‐183 cluster (hsa‐miR‐182‐5p and hsa‐miR‐183‐5p) (Figure 1). There were also differences in the association with disability domains (Figure 3, Table 3) between the miRNAs. This might indicate a difference in function between hsa‐miR‐96‐5p and the remaining two members of the miR‐183 cluster. This hypothesis is further supported by the correlation of both hsa‐miR‐182‐5p and hsa‐miR‐183‐5p with recent relapses and the absence of this correlation when looking at expression of hsa‐miR‐96‐5p (Figure 3), a miRNA previously linked to remission in multiple sclerosis.^50^ Differences in expression levels between members of the same cluster may indicate alterations in miRNAs post‐transcriptionally.^22^

The entire miR‐183 cluster has been previously implicated in multiple sclerosis. In experimental autoimmune encephalomyelitis, an MS mouse model, enhanced expression of the miR‐183 cluster in Th17‐lymphocytes increased the lymphocytes’ production of pathogenic cytokines and autoimmunity through the repression of *FOXO1*.^49^ While this link requires confirmation in human samples, hsa‐miR‐182‐5p was found to be upregulated in the whole blood of paediatric multiple sclerosis patients.^51^ Elevations of hsa‐miR‐96‐5p were also previously observed in remitting multiple sclerosis patient sera^50^ and PBMCs.^52^ Differential expression of miR‐183 cluster miRNAs has not been observed in other leukocytes in multiple sclerosis.^23^

While we did not observe the same subset of differentially expressed erythrocyte miRNAs as in our pilot study, this may be due to cohort differences. The cohort of the small pilot study consisted of stable RRMS patients who were on DMT (predominantly fingolimod or natalizumab).^37^ In contrast, the greatest differences in erythrocyte miRNA expression in this cohort were observed in relapsing multiple sclerosis patients, and all multiple sclerosis patients were either treatment naïve or had not received DMT in the 6 months prior to sample collection. Both natalizumab^53^, ^54^, ^55^ and fingolimod^56^ have been reported to affect erythrocytes. Hence, previously reported erythrocyte miRNAs^37^ may reflect treatment effects.

### 4.2 Potential function of erythrocyte microRNAs

Erythrocyte miRNAs may represent remnants of the precursor transcriptome or they may be involved in intercellular communication through EVs. Having observed an increase in plasma‐borne erythrocyte‐derived EVs during relapse (Supporting Information File 3), we wanted to investigate the latter of the two hypotheses.

Sequencing of erythrocyte‐derived EV miRNAs revealed that miRNAs were selectively packaged into EVs (Figure 4). Selective packaging of EVs in healthy and diseased (cancer) cells *ex vivo* has also been demonstrated in other cell types.^32^ Further, erythrocyte‐derived EV miRNAs demonstrated differential enrichment patterns between multiple sclerosis patients and HCs (Figure 4).

Target genes of miRNAs that were differentially enriched in multiple sclerosis patient EVs compared to those of HCs overlapped with proposed multiple sclerosis susceptibility genes^6^ (Figure 5, Supporting Information File 5). None of the variants, which these susceptibility genes are based on, are sufficient or necessary for the development of multiple sclerosis. Hence, miRNAs may work in concert with single nucleotide polymorphisms (SNPs) to alter expression of susceptibility genes. Increased expression of hsa‐miR‐146a, which was enriched in multiple sclerosis patients’ erythrocyte‐derived EVs (Figure 4, Supporting Information File 4), has been linked to the C allele of rs2910164. This SNP was significantly associated with multiple sclerosis risk.^57^ Hsa‐miR‐146a plays a crucial role in mediating immune responses and inflammation through negative regulation of Toll‐like receptors and inflammatory cytokines.^58^ Increased expression of hsa‐miR‐146a has also been observed in multiple sclerosis patient PBMCs.^59^ Taken together, these results strongly implicate hsa‐miR‐146a in multiple sclerosis risk and pathology.

Several other differentially enriched erythrocyte‐derived EV miRNAs (Figure 4, Supporting Information File 4) have previously been linked to multiple sclerosis. In serum, where blood cell–derived EVs are numerous,^60^ and within circulating EVs, hsa‐miR‐375,^61^, ^62^ hsa‐miR‐342‐3p, hsa‐miR‐374a‐5p,^34^ hsa‐miR‐32‐3p, hsa‐miR‐331‐3p and hsa‐miR‐19a‐3p^61^ were identified as differentially enriched in multiple sclerosis. These reports support that what was observed in our *ex vivo* created erythrocyte‐derived EVs may also occur *in vivo*.

Erythrocyte‐derived EV hsa‐miR‐150‐5p was enriched in multiple sclerosis patients compared to HCs (Figure 4, Supporting Information File 4). Similar trends have been observed in the CSF of patients.^63^ With erythrocyte‐derived EVs being able to cross the BBB,^64^, ^65^ miRNAs within them may contribute to CNS pathology. In fact, silencing or reduction of multiple sclerosis–enriched hsa‐miR‐150‐5p ameliorates experimental autoimmune encephalomyelitis (EAE), a multiple sclerosis mouse model.^66^, ^67^ Further, many of the differentially enriched erythrocyte‐derived EV miRNAs found in serum (hsa‐miR‐375, hsa‐miR‐32‐3p, hsa‐miR‐331‐3p and hsa‐miR‐19a‐3p) correlated with brain lesions and atrophy.^61^

Targets of differentially enriched erythrocyte‐derived EV miRNAs were found to be involved in several developmental pathways of the nervous system as well as histone H3‐K27 demethylation (Table 4). MicroRNAs targeting genes involved in histone H3‐K27 demethylation were found to be reduced in multiple sclerosis patient erythrocyte‐derived EVs. Hence, there may be less translational inhibition of the genes involved in the demethylation pathway and consequently less methylation at lysine 27 of histone H3. This demethylated state of the histone protein facilitates access to the gene promoter region and thereby allows transcription of nearby genes. Acetylation of the same lysine residue has further been shown to enhance gene transcription.^68^ These epigenetic mechanisms have been implicated in Th17 differentiation^69^ and macrophage phenotype.^70^ Both macrophages and Th17 cells are known to be involved in multiple sclerosis pathology.^1^ The H3‐K27 demethylase Kdm6b (gene: *KDM6B*), also known as Jmjd3, was one of the targets of erythrocyte‐derived EV miRNAs that were decreased in multiple sclerosis. Demethylase Jmjd3 was found to be crucial to the development of EAE.^69^ This further supports the involvement of erythrocyte‐derived EV miRNAs in multiple sclerosis pathology.

The erythrocyte miRNA validation study focussed largely on the miR‐183 cluster. While this cluster was not found to be enriched in multiple sclerosis patient erythrocyte‐derived EVs compared to HCs, it may provide some insight as to what is happening in the bone marrow during erythrocyte development. Erythrocytes are part of the myeloid lineage, as are platelets and macrophages.^11^ With erythrocytes being void of nuclei, miRNAs within them may have been transcribed prior to maturation, where they are critical for erythrocyte differentiation.^30^ The miR‐183 cluster has been associated with early development of sensory organs, but also implicated in pathologies such as cancer, neurological diseases (neuropathic pain, nerve injury and neurodegenerative disorders such as multiple‐systems atrophy) and autoimmune conditions (systemic lupus erythematosus and uveal autoimmune genetic syndromes).^22^ Considering the overlap between miR‐183 cluster targets and multiple sclerosis susceptibility genes (Figure 5, Supporting Information File 5), its role in autoimmunity may be affecting other cells of the myeloid lineage during differentiation. While the overlap between miR‐183 cluster targets and multiple sclerosis susceptibility genes suggests some involvement in the disease, further research is required to determine the expression of miR‐183 cluster miRNAs in myeloid precursor cells and its role in multiple sclerosis pathogenesis.

### 4.3 Conclusions and future directions

This study highlights the potential involvement of erythrocyte‐derived EV miRNAs as players in multiple sclerosis pathology and the possibility of developing erythrocyte miRNAs into specific biomarkers for multiple sclerosis. Nonetheless, there are limitations including the small sample size, the use of separate cohorts for the study of erythrocyte and erythrocyte‐derived EV miRNAs and the creation of EVs *ex vivo*. Future studies should aim to address some of the limitations by isolating EVs from plasma, increasing the power of the study to allow for the inclusion of male patients and investigating the functionality of EV miRNAs. This is the first study implicating erythrocyte‐derived EV miRNAs in multiple sclerosis. Potential links between these miRNAs and pathological mechanisms demand further investigation.

## CONFLICT OF INTEREST

Professor J. Lechner‐Scott's institution receives non‐directed funding, as well as honoraria for presentations and membership on advisory boards from Sanofi Genzyme, Biogen, Merck, Teva, Roche and Novartis Australia. V.E. Maltby has received honoraria for presentations from Merck and Biogen.

## AUTHOR CONTRIBUTIONS

Conceptualisation, funding acquisition, methodology, investigation, data curation, formal analysis, visualisation, writing (original draft, reviewing and editing) and project administration: Groen. Conceptualisation, funding acquisition, methodology, writing (reviewing and editing), project administration and supervision: Maltby. Conceptualisation, funding acquisition, methodology, writing (reviewing and editing) and supervision: Scott. Conceptualisation, writing (reviewing and editing), and supervision: Tajouri. Conceptualisation, funding acquisition, writing (reviewing and editing), project administration and supervision: Lechner‐Scott.

## Supporting information

Supplementary file 1: Erythrocyte microRNA sequencing data.

Supplementary file 2: PCR efficiencies for TaqMan Advanced microRNA probes.

Supplementary file 3: Erythrocyte‐derived extracellular vesicles in patient and healthy control plasma.

Supplementary file 4: Erythrocyte‐derived extracellular vesicle microRNAs.

Supplementary file 5: MicroRNA targets and overlap with multiple sclerosis susceptibility genes.

## Data Availability

The data that support the findings of this study are available from the corresponding author upon reasonable request.

## References

[1] Dendrou CA , Fugger L , Friese MA . Immunopathology of multiple sclerosis. Nat Rev Immunol. 2015;15(9):545‐558. 26250739 10.1038/nri3871

[2] Haussleiter IS , Brune M , Juckel G . Psychopathology in multiple sclerosis: diagnosis, prevalence and treatment. Ther Adv Neurol Disord. 2009;2(1):13‐29. 21180640 10.1177/1756285608100325 PMC3002616

[3] Ribbons K , Lea R , Tiedeman C , Mackenzie L , Lechner‐Scott J . Ongoing increase in incidence and prevalence of multiple sclerosis in Newcastle, Australia: a 50‐year study. Mult Scler. 2017;23(8):1063‐1071. 27682228 10.1177/1352458516671819

[4] Simpson S Jr , Wang W , Otahal P , Blizzard L , van der Mei IAF , Taylor BV . Latitude continues to be significantly associated with the prevalence of multiple sclerosis: an updated meta‐analysis. J Neurol Neurosurg Psychiatry. 2019;90(11):1193‐1200. 31217172 10.1136/jnnp-2018-320189

[5] Compston A , Coles A. Multiple sclerosis. Lancet. 2008;372(9648):1502‐1517. 18970977 10.1016/S0140-6736(08)61620-7

[6] International Multiple Sclerosis Genetics Consortium . Multiple sclerosis genomic map implicates peripheral immune cells and microglia in susceptibility. Science. 2019;365(6460):eaav7188. 31604244 10.1126/science.aav7188 PMC7241648

[7] Lublin FD , Reingold SC , Cohen JA , et al. Defining the clinical course of multiple sclerosis: the 2013 revisions. Neurology. 2014;83(3):278‐286. 24871874 10.1212/WNL.0000000000000560 PMC4117366

[8] Cree BAC , Hollenbach JA , Bove R , et al. Silent progression in disease activity‐free relapsing multiple sclerosis. Ann Neurol. 2019;85(5):653‐666. 30851128 10.1002/ana.25463 PMC6518998

[9] Groen K , Maltby VE , Sanders KA , Scott RJ , Tajouri L , Lechner‐Scott J . Erythrocytes in multiple sclerosis–forgotten contributors to the pathophysiology? Mult Scler J Exp Transl Clin. 2016;2 10.1177/2055217316649981 PMC5433403 28607726

[10] Klinken SP. Red blood cells. Int J Biochem Cell Biol. 2002;34(12):1513‐1518. 12379271 10.1016/s1357-2725(02)00087-0

[11] Marieb EN , Hoehn K. Human Anatomy & Physiology. Boston, MA: Pearson; 2013.

[12] Tas SW , Klickstein LB , Barbashov SF , Nicholson‐Weller A . C1q and C4b bind simultaneously to CR1 and additively support erythrocyte adhesion. J Immunol. 1999;163(9):5056‐5063. 10528211

[13] Brownlee WJ , Hardy TA , Fazekas F , Miller DH . Diagnosis of multiple sclerosis: progress and challenges. Lancet. 2017;389(10076):1336‐1346. 27889190 10.1016/S0140-6736(16)30959-X

[14] Thompson AJ , Banwell BL , Barkhof F , et al. Diagnosis of multiple sclerosis: 2017 revisions of the McDonald criteria. Lancet Neurol. 2018;17(2):162‐173. 29275977 10.1016/S1474-4422(17)30470-2

[15] Hu F , Qian ZW. Characteristic analysis of white matter lesions in migraine patients with MRI. Eur Rev Med Pharmacol Sci. 2016;20(6):1032‐1036. 27049253

[16] Biesbroek JM , Weaver NA , Biessels GJ . Lesion location and cognitive impact of cerebral small vessel disease. Clin Sci (Lond). 2017;131(8):715‐728. 28385827 10.1042/CS20160452

[17] Disanto G , Barro C , Benkert P , et al. Serum neurofilament light: a biomarker of neuronal damage in multiple sclerosis. Ann Neurol. 2017;81(6):857‐870. 28512753 10.1002/ana.24954 PMC5519945

[18] Kuhle J , Barro C , Andreasson U , et al. Comparison of three analytical platforms for quantification of the neurofilament light chain in blood samples: ELISA, electrochemiluminescence immunoassay and Simoa. Clin Chem Lab Med. 2016;54(10):1655‐1661. 27071153 10.1515/cclm-2015-1195

[19] Rohrer JD , Woollacott IOC , Dick KM , et al. Serum neurofilament light chain protein is a measure of disease intensity in frontotemporal dementia. Neurology. 2016;87(13):1329‐1336. 27581216 10.1212/WNL.0000000000003154 PMC5047041

[20] Tiedt S , Duering M , Barro C . Serum neurofilament light: a biomarker of neuroaxonal injury after ischemic stroke. Neurology. 2018;91(14):e1338‐e1347. 30217937 10.1212/WNL.0000000000006282

[21] Esteller M. Non‐coding RNAs in human disease. Nat Rev Genet. 2011;12(12):861‐874. 22094949 10.1038/nrg3074

[22] Dambal S , Shah M , Mihelich B , Nonn L . The microRNA‐183 cluster: the family that plays together stays together. Nucleic Acids Res. 2015;43(15):7173‐7188. 26170234 10.1093/nar/gkv703 PMC4551935

[23] Yang X , Wu Y , Zhang B , Ni B . Noncoding RNAs in multiple sclerosis. Clin Epigenetics. 2018;10(1):149. 30497529 10.1186/s13148-018-0586-9 PMC6267072

[24] Goh S‐H , Lee YT , Bouffard GG , Miller JL . Hembase: browser and genome portal for hematology and erythroid biology. Nucleic Acids Res. 2004;32(suppl 1):D572‐D574. 10.1093/nar/gkh129 PMC308863 14681483

[25] Azzouzi I , Moest H , Wollscheid B , Schmugge M , Eekels JJM , Speer O . Deep sequencing and proteomic analysis of the microRNA‐induced silencing complex in human red blood cells. Exp Hematol. 2015;43(5):382‐392. 25681748 10.1016/j.exphem.2015.01.007

[26] Chen S‐Y , Wang Y , Telen MJ , Chi J‐T . The genomic analysis of erythrocyte microRNA expression in sickle cell diseases. PLoS One. 2008;3(6):e2360. 18523662 10.1371/journal.pone.0002360 PMC2408759

[27] Juzenas S , Venkatesh G , Hübentha M , et al. A comprehensive, cell specific microRNA catalogue of human peripheral blood. Nucleic Acids Res. 2017;45(16):9290‐9301. 28934507 10.1093/nar/gkx706 PMC5766192

[28] LaMonte G , Philip N , Reardon J , et al. Translocation of sickle cell erythrocyte microRNAs into *Plasmodium falciparum* inhibits parasite translation and contributes to malaria resistance. Cell Host Microbe. 2012;12(2):187‐199. 22901539 10.1016/j.chom.2012.06.007 PMC3442262

[29] Sun L , Fan F , Li R , et al. Different erythrocyte microRNA profiles in low‐ and high‐altitude individuals. Front Physiol. 2018;9(1099):1099. 30154732 10.3389/fphys.2018.01099 PMC6102482

[30] Listowski MA , Heger E , Bogusławska DM , et al. microRNAs: fine tuning of erythropoiesis. Cell Mol Biol Lett. 2013;18(1):34‐46. 23124859 10.2478/s11658-012-0038-z PMC6276011

[31] Harisa GI , Badran MM , Alanazi FK . Erythrocyte nanovesicles: biogenesis, biological roles and therapeutic approach: erythrocyte nanovesicles. Saudi Pharm J. 2017;25(1):8‐17. 28223857 10.1016/j.jsps.2015.06.010 PMC5310160

[32] Zaborowski MP , Balaj L , Breakefield XO , Lai CP . Extracellular vesicles: composition, biological relevance, and methods of study. Bioscience. 2015;65(8):783‐797. 26955082 10.1093/biosci/biv084 PMC4776721

[33] Mantel P‐Y , Hjelmqvist D , Walch M , et al. Infected erythrocyte‐derived extracellular vesicles alter vascular function via regulatory Ago2‐miRNA complexes in malaria. Nat Commun. 2016;7:12727. 27721445 10.1038/ncomms12727 PMC5062468

[34] Ebrahimkhani S , Vafaee F , Young PE , et al. Exosomal microRNA signatures in multiple sclerosis reflect disease status. Sci Rep. 2017;7(1):14293. 29084979 10.1038/s41598-017-14301-3 PMC5662562

[35] Saenz‐Cuesta M , Osorio‐Querejeta I , Otaegui D . Extracellular vesicles in multiple sclerosis: what are they telling us? Front Cell Neurosci. 2014;8:100. 24734004 10.3389/fncel.2014.00100 PMC3975116

[36] Doss JF , Corcoran DL , Jima DD , Telen MJ , Dave SS , Chi J‐T . A comprehensive joint analysis of the long and short RNA transcriptomes of human erythrocytes. BMC Genomics. 2015;16(1):952. 26573221 10.1186/s12864-015-2156-2 PMC4647483

[37] Groen K , Maltby VE , Lea RA , et al. Erythrocyte microRNA sequencing reveals differential expression in relapsing‐remitting multiple sclerosis. BMC Med Genomics. 2018;11(1):48. 29783973 10.1186/s12920-018-0365-7 PMC5963124

[38] Butzkueven H , Chapman J , Cristiano E , et al. MSBase: an international, online registry and platform for collaborative outcomes research in multiple sclerosis. Mult Scler. 2006;12(6):769‐774. 17263005 10.1177/1352458506070775

[39] Tokar T , Pastrello C , Rossos AEM , et al. mirDIP 4.1—integrative database of human microRNA target predictions. Nucleic Acids Res. 2017;46(D1):D360‐D370. 10.1093/nar/gkx1144 PMC5753284 29194489

[40] Mi H , Muruganujan A , Ebert D , Huang X , Thomas PD . PANTHER version 14: more genomes, a new PANTHER GO‐slim and improvements in enrichment analysis tools. Nucleic Acids Res. 2018;47(D1):D419‐D426. 10.1093/nar/gky1038 PMC6323939 30407594

[41] Cordero F , Beccuti M , Arigoni M , Donatelli S , Calogero RA . Optimizing a massive parallel sequencing workflow for quantitative miRNA expression analysis. PLoS One. 2012;7(2):e31630. 22363693 10.1371/journal.pone.0031630 PMC3282730

[42] Schofield PW , Lee SJ , Lewin TJ , et al. The audio recorded cognitive screen (ARCS): a flexible hybrid cognitive test instrument. J Neurol Neurosurg Psychiatry. 2010;81(6):602‐607. 19965841 10.1136/jnnp.2009.188003

[43] Solomon AJ , Corboy JR. The tension between early diagnosis and misdiagnosis of multiple sclerosis. Nat Rev Neurol. 2017;13:567. 28799551 10.1038/nrneurol.2017.106

[44] Kavaliunas A , Manouchehrinia A , Stawiarz L , et al. Importance of early treatment initiation in the clinical course of multiple sclerosis. Mult Scler. 2017;23(9):1233‐1240. 27754943 10.1177/1352458516675039

[45] Tufekci KU , Oner MG , Genc S , Genc K . MicroRNAs and multiple sclerosis. Autoimmune Dis. 2010;2011:807426. 21188194 10.4061/2011/807426 PMC3003960

[46] Sarachana T , Kulkarni S , Atreya CD . Evaluation of small noncoding RNAs in ex vivo stored human mature red blood cells: changes in noncoding RNA levels correlate with storage lesion events. Transfusion. 2015;55(11):2672‐2683. 26174076 10.1111/trf.13235

[47] Dai R , Zhang Y , Khan D , et al. Identification of a common lupus disease‐associated microRNA expression pattern in three different murine models of lupus. PLoS One. 2010;5(12):e14302. 21170274 10.1371/journal.pone.0014302 PMC3000827

[48] Chen Y‐J , Chang W‐A , Hsu Y‐L , Chen C‐H , Kuo P‐L . Deduction of novel genes potentially involved in osteoblasts of rheumatoid arthritis using next‐generation sequencing and bioinformatic approaches. Int J Mol Sci. 2017;18(11):E2396. 29137139 10.3390/ijms18112396 PMC5713364

[49] Ichiyama K , Gonzalez‐Martin A , Kim B‐S , et al. The MicroRNA‐183‐96‐182 cluster promotes T helper 17 cell pathogenicity by negatively regulating transcription factor Foxo1 expression. Immunity. 2016;44(6):1284‐98. 27332731 10.1016/j.immuni.2016.05.015 PMC4918454

[50] Sedeeq MS , El‐Nahrery EMA , Shalaby N , et al. Micro‐RNA‐96 and interleukin‐10 are independent biomarkers for multiple sclerosis activity. J Neurol Sci. 2019;403:92‐96. 31238191 10.1016/j.jns.2019.06.022

[51] Liguori M , Nuzziello N , Licciulli F , et al. Combined microRNA and mRNA expression analysis in pediatric multiple sclerosis: an integrated approach to uncover novel pathogenic mechanisms of the disease. Hum Mol Genet. 2018;27(1):66‐79. 29087462 10.1093/hmg/ddx385

[52] Otaegui D , Baranzini SE , Armañanzas R , et al. Differential microRNA expression in PBMC from multiple sclerosis patients. PLoS One. 2009;4(7):e6309. 19617918 10.1371/journal.pone.0006309 PMC2708922

[53] Bridel C , Beauverd Y , Samii K , Lalive PH . Hematologic modifications in natalizumab‐treated multiple sclerosis patients: an 18‐month longitudinal study. Neurol Neuroimmunol Neuroinflamm. 2015;2(4):123. 10.1212/NXI.0000000000000123 PMC4476051 26140281

[54] Jing D , Oelschlaegel U , Ordemann R , et al. CD49d blockade by natalizumab in patients with multiple sclerosis affects steady‐state hematopoiesis and mobilizes progenitors with a distinct phenotype and function. Bone Marrow Transplant. 2010;45(10):1489‐1496. 20098455 10.1038/bmt.2009.381

[55] Lesesve J‐F , Debouverie M , Bittencourt MD , Béné M‐C . CD49d blockade by natalizumab therapy in patients with multiple sclerosis increases immature B‐lymphocytes. Bone Marrow Transplant. 2011;46(11):1489‐1491. 21243032 10.1038/bmt.2010.328

[56] Eberhard M , Ferlinz K , Alizzi K , et al. FTY720‐induced suicidal erythrocyte death. Cell Physiol Biochem. 2010;26(4‐5):761‐766. 21063113 10.1159/000322343

[57] Li Y , Du C , Wang W , et al. Genetic association of MiR‐146a with multiple sclerosis susceptibility in the Chinese population. Cell Physiol Biochem. 2015;35(1):281‐291. 25591770 10.1159/000369695

[58] Testa U , Pelosi E , Castelli G , Labbaye C . miR‐146 and miR‐155: two key modulators of immune response and tumor development. Noncoding RNA. 2017;3(3):22. 10.3390/ncrna3030022 PMC5831915 29657293

[59] Waschbisch A , Atiya M , Linker RA , Potapov S , Schwab S , Derfuss T . Glatiramer acetate treatment normalizes deregulated microRNA expression in relapsing remitting multiple sclerosis. PLoS One. 2011;6(9):e24604. 21949733 10.1371/journal.pone.0024604 PMC3174971

[60] Enjeti AK , Ariyarajah A , D'Crus A , Seldon M , Lincz LF . Correlative analysis of nanoparticle tracking, flow cytometric and functional measurements for circulating microvesicles in normal subjects. Thromb Res. 2016;145:18‐23. 27429418 10.1016/j.thromres.2016.06.029

[61] Regev K , Healy BC , Khalid F , et al. Association between serum microRNAs and magnetic resonance imaging measures of multiple sclerosis severity. JAMA Neurol. 2017;74(3):275‐285. 28114622 10.1001/jamaneurol.2016.5197 PMC6014611

[62] Vistbakka J , Elovaara I , Lehtimäki T , Hagman S . Circulating microRNAs as biomarkers in progressive multiple sclerosis. Mult Scler. 2017;23(3):403‐412. 27246141 10.1177/1352458516651141

[63] Bergman P , Piket E , Khademi M , et al. Circulating miR‐150 in CSF is a novel candidate biomarker for multiple sclerosis. Neurol Neuroimmunol Neuroinflamm. 2016;3(3):e219. 27144214 10.1212/NXI.0000000000000219 PMC4841644

[64] Matsumoto J , Stewart T , Banks WA , Zhang J . The transport mechanism of extracellular vesicles at the blood‐brain barrier. Curr Pharm Des. 2017;23(40):6206‐6214. 28914201 10.2174/1381612823666170913164738

[65] Matsumoto J , Stewart T , Sheng L , et al. Transmission of alpha‐synuclein‐containing erythrocyte‐derived extracellular vesicles across the blood‐brain barrier via adsorptive mediated transcytosis: another mechanism for initiation and progression of Parkinson's disease? Acta Neuropathol Commun. 2017;5(1):71. 28903781 10.1186/s40478-017-0470-4 PMC5598000

[66] Al‐Ghezi ZZ , Miranda K , Nagarkatti M , Nagarkatti PS . Combination of cannabinoids, delta9‐tetrahydrocannabinol and cannabidiol, ameliorates experimental multiple sclerosis by suppressing neuroinflammation through regulation of miRNA‐mediated signaling pathways. Front Immunol. 2019;10:1921. 31497013 10.3389/fimmu.2019.01921 PMC6712515

[67] Hu Z , Cui Y , Qiao X , et al. Silencing miR‐150 ameliorates experimental autoimmune encephalomyelitis. Front Neurosci. 2018;12:465. 30050402 10.3389/fnins.2018.00465 PMC6052910

[68] He H , Hu Z , Xiao H , Zhou F , Yang B . The tale of histone modifications and its role in multiple sclerosis. Hum Genomics. 2018;12(1):31. 29933755 10.1186/s40246-018-0163-5 PMC6013900

[69] Liu Z , Cao W , Xu L , et al. The histone H3 lysine‐27 demethylase Jmjd3 plays a critical role in specific regulation of Th17 cell differentiation. J Mol Cell Biol. 2015;7(6):505‐516. 25840993 10.1093/jmcb/mjv022

[70] Ishii M , Wen H , Corsa CAS , et al. Epigenetic regulation of the alternatively activated macrophage phenotype. Blood. 2009;114(15):3244‐3254. 19567879 10.1182/blood-2009-04-217620 PMC2759649

