# Supplementary material for: Erythrocyte microRNAs show biomarker potential and implicate multiple sclerosis susceptibility genes
Source: Clin Transl Med. 2020 Apr 10;10(1):74–90. doi: 10.1002/ctm2.22 (PMC7240864; doi:10.1002/ctm2.22)
Supplement: Supplementary file 1 — Erythrocyte microRNA sequencing data. [file CTM2-10-74-s005.docx]

**Supplementary file 1: Erythrocyte microRNA sequencing data.**


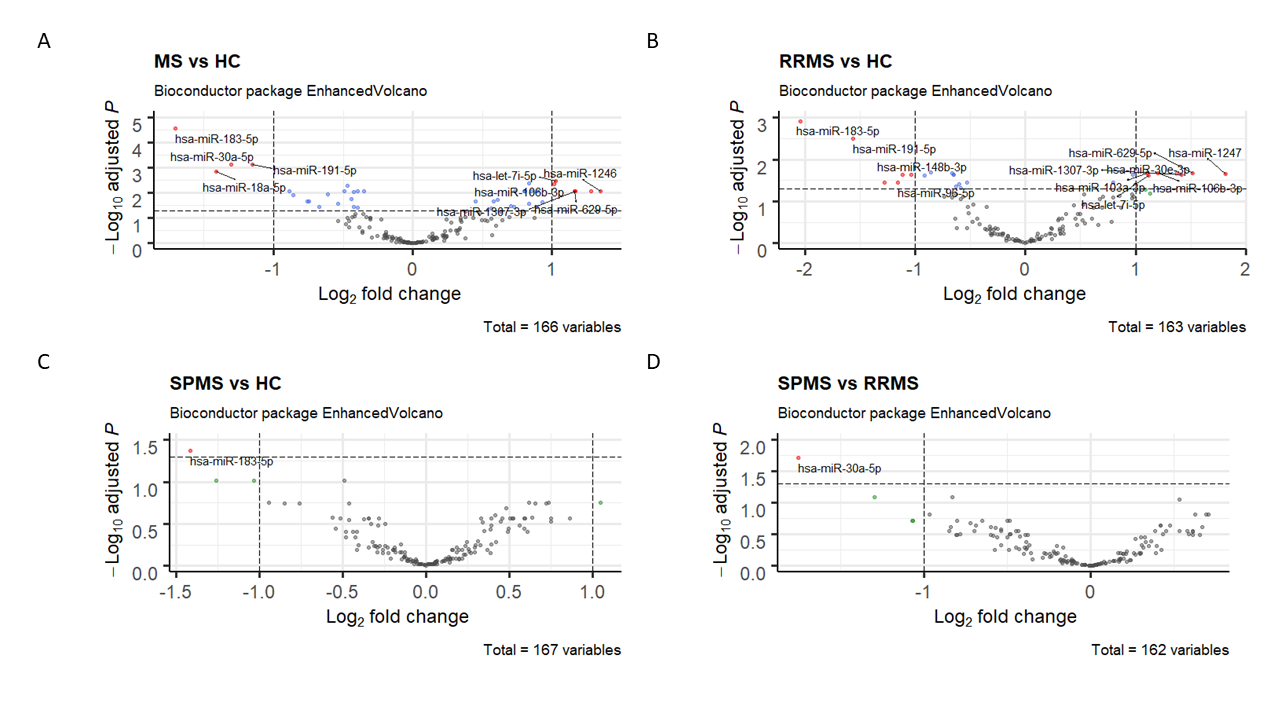


**Supplementary Figure 1 (in colour): Volcano plots of erythrocyte microRNA sequencing results.** Volcano plots of differentially expressed erythrocyte miRNAs, between (A) all MS patients (RRMS and SPMS combined, n = 20) and HCs (n = 18), (B) RRMS patients (n = 9) and HCs (n = 9), (C) SPMS patients (n = 11) and HCs (n = 9) and (D) SPMS patients (n = 11) and RRMS patients (n = 9). Differentially expressed miRNAs are highlighted in red. Dotted lines represent cut-off values: adjusted p-value < 0.05 and fold change ≥ 2 or ≤ 0.5. Erythrocyte miRNAs that were chosen for validation with RT-qPCR are labelled (some labels are missing to avoid overcrowding of the plot). HC – healthy control; miRNA – microRNA; MS – Multiple Sclerosis; RRMS – relapsing-remitting Multiple Sclerosis; RT-qPCR – reverse-transcription quantitative polymerase chain reaction; SPMS – secondary progressive Multiple Sclerosis.

**Supplementary Table 1: Differential expression of erythrocyte microRNAs between different groups.**

| MS vs HC | | | | | | |
| --- | --- | --- | --- | --- | --- | --- |
| microRNA | **baseMean** | **log2FoldChange** | **lfcSE** | **stat** | **p-value** | **padj** |
| hsa-miR-183-5p | 7579 | -1.707 | 0.322 | 5.307 | 1.11E-07 | 2.77E-05 |
| hsa-miR-30a-5p | 79 | -1.304 | 0.292 | 4.468 | 7.89E-06 | 7.48E-04 |
| hsa-miR-191-5p | 32572 | -1.149 | 0.259 | 4.440 | 9.01E-06 | 7.48E-04 |
| hsa-miR-18a-5p | 547 | -1.413 | 0.334 | 4.233 | 2.30E-05 | 1.43E-03 |
| hsa-let-7i-5p | 10010 | 1.028 | 0.258 | -3.984 | 6.77E-05 | 3.37E-03 |
| hsa-miR-106b-3p | 582 | 1.015 | 0.265 | -3.832 | 1.27E-04 | 4.52E-03 |
| hsa-miR-1307-3p | 9 | 1.168 | 0.347 | -3.362 | 7.74E-04 | 8.73E-03 |
| hsa-miR-3960 | 20 | 1.278 | 0.367 | -3.484 | 4.95E-04 | 8.73E-03 |
| hsa-miR-629-5p | 25 | 1.163 | 0.346 | -3.359 | 7.82E-04 | 8.73E-03 |
| hsa-miR-1246 | 24 | 1.346 | 0.377 | -3.575 | 3.50E-04 | 8.73E-03 |
| RRMS vs HC | | | | | | |
| microRNA | **baseMean** | **log2FoldChange** | **lfcSE** | **stat** | **p-value** | **padj** |
| hsa-miR-183-5p | 2015 | -2.042 | 0.461 | -4.428 | 9.50E-06 | 1.23E-03 |
| hsa-miR-191-5p | 12775 | -1.567 | 0.386 | -4.061 | 4.89E-05 | 3.18E-03 |
| hsa-miR-629-5p | 71 | 1.515 | 0.447 | 3.390 | 6.99E-04 | 2.15E-02 |
| hsa-miR-30e-3p | 92 | 1.371 | 0.421 | 3.252 | 1.14E-03 | 2.15E-02 |
| hsa-miR-106b-3p | 1366 | 1.202 | 0.374 | 3.211 | 1.32E-03 | 2.15E-02 |
| hsa-miR-103a-3p | 5283 | 1.106 | 0.331 | 3.340 | 8.38E-04 | 2.15E-02 |
| hsa-miR-1246 | 68 | 1.812 | 0.572 | 3.169 | 1.53E-03 | 2.21E-02 |
| hsa-miR-1307-3p | 25 | 1.418 | 0.458 | 3.098 | 1.95E-03 | 2.27E-02 |
| hsa-miR-501-3p | 63 | -1.039 | 0.344 | -3.021 | 2.52E-03 | 2.27E-02 |
| hsa-miR-148b-3p | 154 | -1.116 | 0.364 | -3.066 | 2.17E-03 | 2.27E-02 |
| hsa-let-7i-5p | 23186 | 1.114 | 0.377 | 2.957 | 3.11E-03 | 2.38E-02 |
| hsa-miR-96-5p | 103 | -1.153 | 0.417 | -2.765 | 5.69E-03 | 3.57E-02 |
| SPMS vs HC | | | | | | |
| microRNA | **baseMean** | **log2FoldChange** | **lfcSE** | **stat** | **p-value** | **padj** |
| hsa-miR-183-5p | 2770 | -1.417 | 0.386 | -3.676 | 2.37E-04 | 4.29E-02 |
| SPMS vs RRMS | | | | | | |
| microRNA | **baseMean** | **log2FoldChange** | **lfcSE** | **stat** | **p-value** | **padj** |
| hsa-miR-30a-5p | 52 | -1.754 | 0.446 | -3.935 | 8.34E-05 | 1.93E-02 |

baseMean – mean normalised read counts in the reference group (HC or RRMS for RRMS vs SPMS comparison); HC – healthy control; lfcSE – log2(fold change) standard error; MS – Multiple Sclerosis; padj – false discovery rate-adjusted p-values; RRMS – relapsing-remitting Multiple Sclerosis; SPMS – secondary progressive Multiple Sclerosis.
