# Supplementary material for: Erythrocyte microRNAs show biomarker potential and implicate multiple sclerosis susceptibility genes
Source: Clin Transl Med. 2020 Apr 10;10(1):74–90. doi: 10.1002/ctm2.22 (PMC7240864; doi:10.1002/ctm2.22)
Supplement: Supplementary file 2 — PCR efficiencies for TaqMan Advanced microRNA probes. [file CTM2-10-74-s004.docx]

**Supplementary file 2: PCR efficiencies for TaqMan Advanced microRNA probes.**

**Supplementary Table 2: PCR Efficiencies.**

| Probe ID | PCR Efficiency (%) |
| --- | --- |
| hsa-miR-96-5p (478215_mir) | 90.89 |
| hsa-miR-18a-5p (478551_mir) | 97.11 |
| hsa-let-7i-5p (478375_mir) | 102.99 |
| hsa-miR-183-5p (477937_mir) | 101.34 |
| hsa-miR-30a-5p (479448_mir) | 113.58 |
| hsa-miR-106b-3p (477866_mir) | 96.63 |
| hsa-miR-191-5p (477952_mir) | 97.90 |
| hsa-miR-103a-3p (478253_mir) | 90.60 |
| hsa-miR-629-5p (478183_mir) | 99.06 |
| hsa-miR-30e-3p (478388_mir) | 90.69 |
| hsa-miR-1307-3p (483036_mir) | 102.08 |
| hsa-miR-1246 (477881_mir) | undetermined |
| hsa-miR-182-5p (477935_mir) | 103.03 |
| hsa-miR-152-3p (477921_mir) | 93.74 |

PCR efficiencies were determined using a serial dilution of the cDNA from three different samples of the discovery cohort.
