# Supplementary material for: Erythrocyte microRNAs show biomarker potential and implicate multiple sclerosis susceptibility genes
Source: Clin Transl Med. 2020 Apr 10;10(1):74–90. doi: 10.1002/ctm2.22 (PMC7240864; doi:10.1002/ctm2.22)
Supplement: Supplementary file 3 — Erythrocyte‐derived extracellular vesicles in patient and healthy control plasma. [file CTM2-10-74-s003.docx]

**Supplementary file 3: Erythrocyte-derived extracellular vesicles in patient and healthy control plasma.**

**Methods:**

The plasma cohort consisted of 17 stable RRMS patients, 13 relapsing Multiple Sclerosis patients, 9 SPMS patients and 27 HCs (Supplementary Table 3).

Platelet-free plasma was obtained via a series differential centrifugation steps and stored at -80°C until flow cytometry staining.

Ten µl of platelet-free plasma were stained with APC-conjugated anti-CD235a antibody (Clone GA-R2, HIR2, BD Pharmingen, USA). Samples were run on a modified LSRFortessa (BD Biosciences, USA) with 0.1 µm sheath fluid filter. Staining buffer was filtered with 0.1 µm filters. Size gates were set using flow cytometer calibration and size reference beads (Invitrogen, USA). An EV gate was set for events that were ˂ 1 µm. Plasma EVs were quantified using CountBright beads (Invitrogen, USA). Negative controls (staining buffer and antibodies only, no sample) were run with every batch of samples. Plasma EV concentrations were calculated as follows:

$$plasma EVs (per \mu l) = \left( \left( \left( \frac{\#EVs}{\#beads} * b \right)* dilution factor \right)-a \right)* \left( 1-AF \right)$$

$$b(bead concentration in sample)=\frac{bead concentration * bead volume}{total volume}$$

$$a\left( antibody EVs \right)=\left( \frac{\#EVs}{\#beads} * b \right)* dilution factor$$

$$AF - autofluorescence$$

Statistical significance was determined through two-tailed unpaired student’s t tests.

**Results:**

Cohort demographics are summarised in Supplementary Table 3. Plasma-borne erythrocyte-derived EVs were increased in relapse compared to HCs (p < 0.05) (Supplementary Figure 2).

**Supplementary Table 3: Plasma extracellular vesicle cohort.**

| Plasma cohort | | | | | |
| --- | --- | --- | --- | --- | --- |
|  | RRMS (stable) | Relapse | HC (RRMS) | SPMS | HC (SPMS) |
| n | 17 | 13 | 17 | 9 | 10 |
| Age (years) | 51.0 (±12.2) | 38.0 (±12.0) | 50.6 (±11.9) | 59.4 (±9.7) | 60.0 (±6.5) |
| Bench time (hours) | 2.9 (±1.3) | 3.4 (±1.2) | 2.2 (±1.4) | 2.4 (±1.1) | 1.8 (±1.3) |
| Disease duration (years) | 14.7 (±10.7) | 6.0 (±6.5) | n/a | 26.9 (±14.2) | n/a |
| Progression duration (years) | n/a | n/a |  | 13.0 (±17.4) |  |
| EDSS score | 1.6 (±1.0) | 3.0 (±1.8) |  | 5.7 (±2.2) |  |
| Age at onset (years) | 34.8 (±10.4) | 30.2 (±10.7) |  | 29.1 (±15.5) |  |
| Number of relapses | 4 (±3) | 4 (±5) |  | 5 (±4) |  |
| Steroids ≤ 3months (% yes) | 0 | 46.2 |  | 0 |  |
| Days since last relapse | 1510 (±1614) | 18 (±15) |  | 5052 (±5759) |  |

Data shown as mean (± SD). *ARCS scores were only reported if patients had performed an ARCS within 12 months of sample collection. ARCS – audio-recorded cognitive screen; EDSS – expanded disability status scale; HC – healthy control; RRMS – relapsing-remitting Multiple Sclerosis; SD – standard deviation of the mean; SPMS – secondary progressive Multiple Sclerosis; n/a – not applicable.


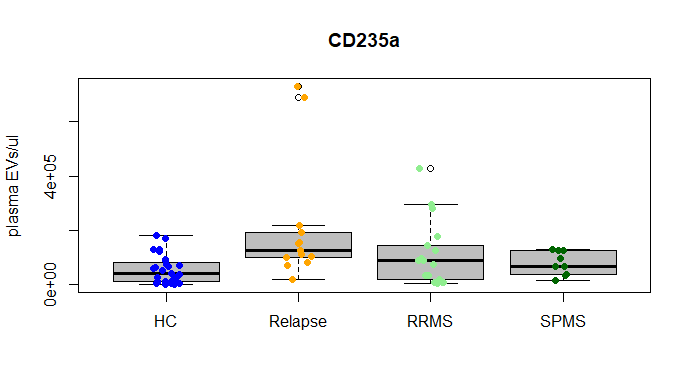


*

**Supplementary Figure 2 (in colour): Plasma-borne CD235a positive EVs.** Erythrocyte-derived EVs (CD235a poistive) per 1 µl plasma in HCs (n = 27, blue), relapsing patients (n = 13, organe), stable RRMS patients (n = 17, light green) and SPMS patients (n = 9, dark green). White circles represent outliers defined as deviating ≥ 1.5-fold from the upper/lower quartile. EVs – extracellular vesicles, HC – healthy control; RRMS – relapsing-remitting Multiple Sclerosis; SPMS – secondary progressive Multiple Sclerosis; * – p<0.05.
