# Supplementary material for: Erythrocyte microRNAs show biomarker potential and implicate multiple sclerosis susceptibility genes
Source: Clin Transl Med. 2020 Apr 10;10(1):74–90. doi: 10.1002/ctm2.22 (PMC7240864; doi:10.1002/ctm2.22)
Supplement: Supplementary file 4 — Erythrocyte‐derived extracellular vesicle microRNAs. [file CTM2-10-74-s002.docx]

**Supplementary file 4: Erythrocyte-derived extracellular vesicle microRNAs.**

| Supplementary Table 4: Differentially enriched erythrocyte-derived extracellular vesicle microRNAs. | | | | | | |
| --- | --- | --- | --- | --- | --- | --- |
| MS vs HC (34 miRNAs) | | | | | | |
|  | baseMean | log2FoldChange | FC | lfcSE | p-value | padj |
| hsa-miR-375 | 151 | -3.80 | 0.07 | 0.59 | 1.78E-13 | 9.01E-11 |
| hsa-miR-581 | 155 | -6.96 | 0.01 | 0.72 | 4.79E-11 | 1.21E-08 |
| hsa-miR-548f-5p | 36 | -1.82 | 0.28 | 0.41 | 6.24E-07 | 0.00011 |
| hsa-miR-659-5p | 79 | -3.85 | 0.07 | 0.50 | 1.95E-06 | 0.00016 |
| hsa-miR-150-5p | 657 | 1.32 | 2.49 | 0.31 | 1.56E-06 | 0.00016 |
| hsa-miR-146a-5p | 1162 | 1.28 | 2.43 | 0.30 | 1.68E-06 | 0.00016 |
| hsa-miR-6857-3p | 59 | -1.18 | 0.44 | 0.28 | 2.76E-06 | 0.0002 |
| hsa-miR-4459 | 176 | -2.39 | 0.19 | 0.60 | 5.52E-06 | 0.00035 |
| hsa-miR-342-3p | 808 | 1.21 | 2.31 | 0.32 | 1.35E-05 | 0.00076 |
| hsa-miR-6802-3p | 32 | -2.96 | 0.13 | 0.49 | 2.51E-05 | 0.00106 |
| hsa-miR-3940-3p | 97 | -3.53 | 0.09 | 0.49 | 2.33E-05 | 0.00106 |
| hsa-miR-619-5p | 20 | -1.04 | 0.49 | 0.29 | 3.71E-05 | 0.00145 |
| hsa-miR-1249-3p | 39 | -1.74 | 0.30 | 0.51 | 5.08E-05 | 0.00184 |
| hsa-miR-5696 | 19 | -1.07 | 0.47 | 0.31 | 5.69E-05 | 0.00192 |
| hsa-miR-548d-3p | 13 | -1.79 | 0.29 | 0.54 | 7.57E-05 | 0.00226 |
| hsa-miR-1273g-3p | 48 | -1.28 | 0.41 | 0.38 | 7.49E-05 | 0.00226 |
| hsa-miR-6881-3p | 50 | -1.59 | 0.33 | 0.48 | 8.40E-05 | 0.00236 |
| hsa-miR-4488 | 53 | -3.05 | 0.12 | 0.40 | 8.92E-05 | 0.00238 |
| hsa-miR-616-5p | 33 | -1.22 | 0.43 | 0.40 | 0.00022 | 0.00565 |
| hsa-miR-877-5p | 36 | -1.82 | 0.28 | 0.62 | 0.00025 | 0.00601 |
| hsa-miR-4707-3p | 191 | -1.35 | 0.39 | 0.46 | 0.00031 | 0.00704 |
| hsa-miR-3157-3p | 10 | -1.13 | 0.46 | 0.41 | 0.00064 | 0.01249 |
| hsa-miR-222-5p | 18 | -1.04 | 0.49 | 0.39 | 0.00092 | 0.0157 |
| hsa-miR-597-3p | 14 | -1.09 | 0.47 | 0.42 | 0.0011 | 0.01694 |
| hsa-miR-4714-5p | 22 | -2.92 | 0.13 | 0.47 | 0.00114 | 0.01703 |
| hsa-miR-1229-3p | 9 | -1.05 | 0.48 | 0.42 | 0.00129 | 0.01812 |
| hsa-miR-4635 | 77 | -1.10 | 0.47 | 0.47 | 0.00191 | 0.02271 |
| hsa-miR-4792 | 47 | -1.09 | 0.47 | 0.48 | 0.00247 | 0.02725 |
| hsa-miR-6894-3p | 20 | -1.18 | 0.44 | 0.46 | 0.0026 | 0.02748 |
| hsa-miR-636 | 51 | -1.90 | 0.27 | 0.52 | 0.00402 | 0.03455 |
| hsa-miR-6877-5p | 33 | -1.39 | 0.38 | 0.38 | 0.00497 | 0.0381 |
| hsa-miR-3691-5p | 22 | -1.02 | 0.49 | 0.54 | 0.00584 | 0.03998 |
| hsa-miR-3661 | 91 | -1.15 | 0.45 | 0.67 | 0.00633 | 0.04113 |
| hsa-miR-1234-3p | 26 | -1.03 | 0.49 | 0.58 | 0.00717 | 0.04312 |
|  |  |  |  |  |  |  |
| RRMS vs HC (17 miRNAs) | | | | | | |
|  | baseMean | log2FoldChange | FC | lfcSE | p-value | padj |
| hsa-miR-375 | 276 | -3.75 | 0.07 | 0.78 | 1E-10 | 4.8E-08 |
| hsa-miR-581 | 155 | -6.98 | 0.01 | 0.92 | 4.4E-08 | 1E-05 |
| hsa-miR-3940-3p | 146 | -3.29 | 0.10 | 0.64 | 6.6E-06 | 0.00106 |
| hsa-miR-627-5p | 21 | -1.34 | 0.39 | 0.40 | 4.3E-05 | 0.00407 |
| hsa-miR-150-5p | 657 | 1.25 | 2.38 | 0.37 | 6E-05 | 0.00409 |
| hsa-miR-548f-5p | 36 | -1.70 | 0.31 | 0.53 | 5.7E-05 | 0.00409 |
| hsa-miR-659-5p | 77 | -3.95 | 0.06 | 0.60 | 0.00016 | 0.0094 |
| hsa-miR-4459 | 176 | -2.20 | 0.22 | 0.82 | 0.00019 | 0.00993 |
| hsa-miR-548d-3p | 13 | -2.01 | 0.25 | 0.73 | 0.00021 | 0.00993 |
| hsa-miR-1249-3p | 39 | -1.70 | 0.31 | 0.69 | 0.00047 | 0.02031 |
| hsa-miR-3173-5p | 20 | -1.11 | 0.46 | 0.43 | 0.00057 | 0.02254 |
| hsa-miR-4488 | 53 | -3.05 | 0.12 | 0.50 | 0.00102 | 0.02579 |
| hsa-miR-5696 | 19 | -1.02 | 0.49 | 0.41 | 0.00077 | 0.02579 |
| hsa-miR-6881-3p | 50 | -1.52 | 0.35 | 0.66 | 0.00075 | 0.02579 |
| hsa-miR-6802-3p | 32 | -2.78 | 0.15 | 0.63 | 0.00097 | 0.02579 |
| hsa-miR-6877-5p | 41 | -1.23 | 0.43 | 0.57 | 0.00133 | 0.03025 |
| hsa-miR-877-5p | 36 | -1.60 | 0.33 | 0.94 | 0.0021 | 0.04363 |
| SPMS vs HC (15 miRNAs) | | | | | | |
|  | baseMean | log2FoldChange | FC | lfcSE | p-value | padj |
| hsa-miR-375 | 276 | -3.16 | 0.11 | 1.04 | 2.8E-10 | 1.2E-07 |
| hsa-miR-3940-3p | 146 | -3.33 | 0.10 | 0.80 | 1.4E-06 | 0.0002 |
| hsa-miR-581 | 155 | -6.68 | 0.01 | 1.14 | 8.5E-06 | 0.00093 |
| hsa-miR-659-5p | 77 | -3.32 | 0.10 | 0.75 | 6E-05 | 0.00438 |
| hsa-miR-636 | 45 | -1.47 | 0.36 | 0.83 | 0.00029 | 0.01712 |
| hsa-miR-144-3p | 1565 | 1.43 | 2.70 | 0.50 | 0.00043 | 0.02045 |
| hsa-miR-374a-5p | 510 | 1.20 | 2.30 | 0.43 | 0.00053 | 0.02097 |
| hsa-miR-19a-3p | 294 | 1.17 | 2.25 | 0.42 | 0.00064 | 0.02349 |
| hsa-miR-6857-3p | 59 | -1.03 | 0.49 | 0.43 | 0.00121 | 0.03789 |
| hsa-miR-548f-5p | 36 | -1.43 | 0.37 | 0.70 | 0.0013 | 0.03789 |
| hsa-miR-144-5p | 3648 | 1.11 | 2.17 | 0.44 | 0.00138 | 0.03789 |
| hsa-miR-183-5p | 2810 | 1.51 | 2.85 | 0.64 | 0.00152 | 0.03903 |
| hsa-miR-6894-3p | 27 | -1.51 | 0.35 | 0.92 | 0.00223 | 0.04983 |
| hsa-miR-874-5p | 31 | -1.15 | 0.45 | 0.58 | 0.00239 | 0.04983 |
| hsa-miR-146a-5p | 1162 | 1.00 | 2.00 | 0.43 | 0.00239 | 0.04983 |
| Relapse vs HC (36 miRNAs) | | | | | | |
|  | baseMean | log2FoldChange | FC | lfcSE | p-value | padj |
| hsa-miR-375 | 276 | -3.79 | 0.07 | 0.99 | 3.9E-12 | 2E-09 |
| hsa-miR-3940-3p | 146 | -3.65 | 0.08 | 0.79 | 2.2E-07 | 3.7E-05 |
| hsa-miR-1224-5p | 82 | -1.71 | 0.31 | 0.39 | 6E-07 | 7.6E-05 |
| hsa-miR-627-3p | 48 | -1.99 | 0.25 | 0.49 | 1.7E-06 | 0.00018 |
| hsa-miR-4521 | 19 | -2.18 | 0.22 | 0.56 | 4.4E-06 | 0.00037 |
| hsa-miR-581 | 155 | -6.66 | 0.01 | 1.14 | 9.1E-06 | 0.00048 |
| hsa-miR-6857-3p | 59 | -1.57 | 0.34 | 0.42 | 8.6E-06 | 0.00048 |
| hsa-miR-636 | 45 | -1.80 | 0.29 | 0.77 | 1E-05 | 0.00048 |
| hsa-miR-331-3p | 39 | -1.38 | 0.39 | 0.57 | 8.9E-06 | 0.00048 |
| hsa-miR-146a-5p | 1162 | 1.64 | 3.12 | 0.42 | 1E-05 | 0.00048 |
| hsa-miR-659-5p | 77 | -3.92 | 0.07 | 0.74 | 1.4E-05 | 0.0006 |
| hsa-miR-150-5p | 657 | 1.58 | 2.99 | 0.43 | 2.8E-05 | 0.00109 |
| hsa-miR-4707-3p | 191 | -2.20 | 0.22 | 0.70 | 4.4E-05 | 0.00161 |
| hsa-miR-548ay-3p | 34 | -1.22 | 0.43 | 0.36 | 5.8E-05 | 0.00197 |
| hsa-miR-148a-5p | 60 | 1.14 | 2.20 | 0.33 | 9.9E-05 | 0.00315 |
| hsa-miR-342-3p | 808 | 1.49 | 2.81 | 0.45 | 0.00011 | 0.00318 |
| hsa-miR-3665 | 27 | -1.87 | 0.27 | 0.63 | 0.00012 | 0.00336 |
| hsa-miR-146b-5p | 371 | 1.18 | 2.26 | 0.36 | 0.00014 | 0.00386 |
| hsa-miR-548f-5p | 36 | -1.77 | 0.29 | 0.66 | 0.00026 | 0.00619 |
| hsa-miR-6741-3p | 14 | -1.09 | 0.47 | 0.39 | 0.00044 | 0.00977 |
| hsa-miR-4301 | 213 | -1.32 | 0.40 | 0.50 | 0.00044 | 0.00977 |
| hsa-miR-4459 | 176 | -2.16 | 0.22 | 1.13 | 0.0008 | 0.01625 |
| hsa-miR-6802-3p | 32 | -3.09 | 0.12 | 0.78 | 0.0009 | 0.01692 |
| hsa-miR-619-5p | 20 | -1.13 | 0.46 | 0.44 | 0.00088 | 0.01692 |
| hsa-miR-1273g-3p | 48 | -1.40 | 0.38 | 0.62 | 0.00106 | 0.01913 |
| hsa-miR-155-5p | 452 | 1.17 | 2.25 | 0.45 | 0.00116 | 0.02023 |
| hsa-miR-4714-5p | 22 | -3.45 | 0.09 | 0.72 | 0.00135 | 0.02274 |
| hsa-miR-1249-3p | 39 | -1.66 | 0.32 | 0.91 | 0.00171 | 0.02707 |
| hsa-miR-6894-3p | 27 | -1.55 | 0.34 | 0.88 | 0.00219 | 0.03365 |
| hsa-miR-6866-5p | 31 | -1.04 | 0.48 | 0.49 | 0.00284 | 0.04239 |
| hsa-miR-32-3p | 14 | -1.06 | 0.48 | 0.52 | 0.00302 | 0.04371 |
| hsa-miR-3157-3p | 10 | -1.27 | 0.42 | 0.68 | 0.00331 | 0.04669 |
| hsa-miR-6783-3p | 20 | -1.09 | 0.47 | 0.55 | 0.00371 | 0.04755 |
| hsa-miR-6861-5p | 23 | -1.12 | 0.46 | 0.58 | 0.00354 | 0.04755 |
| hsa-miR-874-5p | 31 | -1.09 | 0.47 | 0.56 | 0.00375 | 0.04755 |
| hsa-miR-616-5p | 33 | -1.17 | 0.44 | 0.67 | 0.00408 | 0.04925 |
| Relapse vs RRMS (1 miRNA) | | | | | | |
|  | baseMean | log2FoldChange | FC | lfcSE | p-value | padj |
| hsa-miR-148a-5p | 60 | 1.39 | 2.62 | 0.36 | 6.3E-06 | 0.00321 |

There were no differentially expressed miRNAs between SPMS and RRMS. baseMean - mean normalised sequencing counts in the reference group (HC or RRMS); FC – fold change; HC – healthy control; lfcSE – log2(fold change) standard error; miRNA – microRNA; MS – Multiple Sclerosis; padj – false discovery rate-adjusted p-value; RRMS – relapsing-remitting Multiple Sclerosis; SPMS – secondary progressive Multiple Sclerosis.
