# Supplementary material for: Erythrocyte microRNAs show biomarker potential and implicate multiple sclerosis susceptibility genes
Source: Clin Transl Med. 2020 Apr 10;10(1):74–90. doi: 10.1002/ctm2.22 (PMC7240864; doi:10.1002/ctm2.22)
Supplement: Supplementary file 5 — MicroRNA targets and overlap with multiple sclerosis susceptibility genes. [file CTM2-10-74-s001.docx]

**Supplementary file 5: MicroRNA targets and overlap with Multiple Sclerosis susceptibility genes.**

**Supplementary Table 5: MicroRNA targets and Multiple Sclerosis susceptibility genes in overlapping Venn diagram fields (see Figure 5D).**

1 – miR-183 cluster; 2 – MS susceptibility genes; 3 – erythrocyte-derived EV miRNAs down; 4 – erythrocyte-derived EV miRNAs up

| Groups | Total | Target |
| --- | --- | --- |
| 1 2 3 4 | 10 | *MYB, LPP, FRMD6, HOXA1, FAM76B, JADE2, ATXN1, BACH2, AFF1, FOXP1* |
| 1 2 3 | 16 | *ZFP36L1, TMEM50B, NCOA1, ACTR2, RAPH1, RRAS2, LEF1, SLC30A7, BCL6, TGFBR3, ADAT2, EVI5, RND3, MYO19, TET2, HBS1L* |
| 1 2 4 | 18 | *ERG, CD28, CLEC2D, CD226, JAZF1, RREB1, TP53INP1, SLC12A5, STK38, KCNN3, PHACTR2, SYPL1, MTCH2, CLCN6, ZC3HAV1, SESN3, DAB2, TNFAIP8* |
| 1 3 4 | 449 | *SAMD4A, KCNMA1, APBB2, LDB3, GDA, MAP3K3, OSBPL8, IPO9, TEAD1, TIGAR, PHF20, RAD23B, FRS2, RNF4, TPM3, SYT13, LCOR, OPHN1, MMP16, CAMTA1, LIMD1, RICTOR, IRS1, CDK17, SDC2, USP32, QKI, NCAM1, GMFB, JADE1, MOB1B, DCX, GPR158, LIN28B, TMED7, ELAVL2, CPD, EIF2S1, AKAP2, XIAP, SLC16A2, NOTCH2, NFAT5, FBXL5, BSDC1, MAP2K4, ZBTB41, VAT1, WDR26, NR4A3, MAGI1, SRSF3, MAPK1IP1L, ZDHHC17, CACNA1C, MAN1A1, RMND5A, CCDC117, NTRK2, PFKFB2, BIRC6, TACC1, CYB5B, RAB6A, CHD2, CREB1, CFL2, TBL1XR1, RPRD1A, CPEB4, CAMSAP2, C16orf72, DIO2, NUMB, PGR, SOX6, ZNF532, AZIN1, NHS, RAPGEF5, ZMYM2, UNC5D, STAM2, MAF, PDGFRA, DMD, NOVA1, FZD3, FNDC3B, RIMKLA, TLE4, JPH1, LIN7C, LIN28A, SLC4A4, RHOBTB1, DTNA, EEA1, STARD7, RYBP, PSD3, KLF3, ERBB4, WWC2, NUFIP2, AP1G1, GNAI3, ZNF248, PTPRE, ATAD2B, MECP2, ARHGEF12, CDK12, PAFAH1B1, MYO6, MYO1C, PRKCI, ARFGEF3, IPO8, PHC3, DCAF12, SLCO3A1, SH3GLB1, SLC12A6, PAG1, ANKRD28, DYNC1LI2, ZNF609, TCF12, ARMC8, AEBP2, SMC1A, RCC2, EGR1, DYRK2, PCLO, PRPF4B, KIAA1217, STC1, ANK2, ARPP19, SLC1A2, CGGBP1, PCDHA9, NAA50, PHF19, GRIN2B, ZNF518A, ROBO1, GID4, EBF3, EIF5A2, TGFBR1, TUB, KLF12, DENND5B, NMT2, SLC4A7, SGMS1, CAMK2N1, SLITRK6, LASP1, EGR3, RAP2B, PRICKLE2, SF1, ZCCHC14, WDR47, PKN2, AGO1, PRKCE, CREB3L2, PRKAA2, IVNS1ABP, FOSL2, NCS1, BMPR2, USP31, CUL5, SOX5, DSCAM, COL4A4, SP1, SLC9A6, MBNL1, TBR1, PLAG1, MED1, PHYHIPL, IGF1R, SNX30, EFNB2, PATZ1, TMCC1, DCP2, KMT2A, XBP1, TMX1, MEAF6, ACVR2B, ACVR1B, INSR, MMD, PHF20L1, SNX1, SETD7, GPM6B, PTP4A1, MAP4K4, STX16, PEG10, TRPS1, CASP2, ADAMTS5, DDX3X, PHTF2, KLHL3, INPP5A, IGF2BP1, ZNRF1, KLHL24, CNOT6L, SLC35B4, UBE2G1, USP47, HCN1, PAPOLA, DKK2, PCGF3, TENM4, INTS6, MAP3K20, GLE1, SRSF6, POLE3, SLC6A6, PPP1R9A, ERLIN2, CELF1, INO80D, ABI2, MOB4, CNOT6, PRRG1, TCF4, TIAM1, ACVR2A, SCN1A, ZC3H6, NUAK1, RALGPS1, CCDC6, VTI1A, ASAP1, NHLH2, RIMBP2, SNX13, TEX2, NAV2, REPS2, ITGA6, RUNX1T1, TOX, PDE4D, PRKACB, CLLU1, SLC7A11, VPS53, SLC5A3, FRMD4A, ZC3H12C, YES1, FUT8, BEND4, FBXO30, NR2F2, DTX4, TNRC6B, SIKE1, SMAD2, EFNA5, TNRC6A, PPP3R1, CCNJ, TNFSF11, PGAP1, AMMECR1L, SLC35A2, PID1, TMBIM6, PDE7A, XPNPEP3, LRP2, MTDH, UXS1, SEMA6D, RSBN1, GLDC, SUZ12, KCMF1, SELENOI, CELF2, PDXDC1, ZNF507, CSNK1G1, PCDH17, PHF8, GPR85, SHROOM4, ZFAND5, KIAA1211, CTNND1, GABRG2, ELAVL1, RASSF8, CD47, RAP2C, CLDN1, CD164, GFRA1, SOBP, FAM117B, PLPPR4, APC, SEC24A, TRMT1L, AKAP11, AMOT, BHLHE41, EPM2AIP1, EDEM3, ACSL4, BRWD1, DCAF17, WASL, ADAMTS18, RAC1, ZNF189, ZBTB34, RAB8B, SAMD12, SLC2A13, PHF3, ZNF148, GAN, MARK1, LDLRAD4, CADM2, MEF2A, CAMK2D, NRP2, ARID5B, ATP7A, PPP2R1B, JAG1, PAPPA, NRAS, FBXW2, C1orf21, PBRM1, ZCCHC24, USP9X, PARP11, TENM1, CDK6, AFF4, RIMS3, BZW1, PRKAR1A, ZFP30, NSL1, NOX4, PDS5B, PLEKHA1, TRIM9, VAV2, CSNK1A1, SPIN1, FREM2, TBX15, SNTB2, KPNA1, HMGA2, ETF1, SEL1L, ROCK1, WBP1L, TBC1D4, DNM3, SYT1, SREK1IP1, SS18, IKZF2, AFF2, PTPRD, MLLT1, METTL9, PHF6, PCGF5, WIPF2, PPM1B, CBL, ZBTB33, MXI1, MTURN, MAP3K2, FAM46A, CALU, GNAQ, SCARB2, ADCY6, DST, ERP44, ZEB2, CYLD, WTAP, GABRA4, PDE11A, CADM1, KLHDC10, MAP1B, CREBRF, CTBP2, RAD51, DYRK1A, BTBD7, CCND2, KCTD16, PEX19, YAP1, DLGAP2, SOX11, BCL11A, EIF4G2, GATAD2B, ONECUT2, FAM110B, CAST, DGCR2, VSIG10, POC1B, RFTN2, NPAS3, PRKCA, HIPK1, MAL2, RPS6KA3, LANCL1, RNF169* |
| 2 3 4 | 7 | *ITSN1, ZMIZ1, ZNF365, HIF3A, CAMLG, NPEPPS, JARID2* |
| 1 2 | 33 | *DLEU7, GRB2, RGS14, IL15RA, RASA2, ELMO1, TOX2, DEXI, MAST3, MAP3K14, LRRC31, VANGL2, FAM69A, GPR137, PPM1F, B4GALNT1, FUCA2, NFKBIZ, UBE2D3, EOMES, UBASH3B, CEP85L, NFKB1, BCL10, GLB1, IKZF1, LCK, TBKBP1, THSD7B, DPH5, RAVER2, SLC9A8, L3MBTL3* |
| 1 3 | 681 | *RALYL, EPB41L4B, MIER1, XK, INIP, ING3, TNS3, LHFPL2, LPAR4, USP6NL, ISL1, RBM17, VGLL3, PPIL1, CNGA3, VAT1L, FBXO11, N4BP2L2, HS2ST1, CHMP1B, PPM1A, TOX3, CDC42BPA, SPTBN1, GNA11, GRIK2, GNRHR, UNC13B, KDM6A, AHCYL2, GNG12, UBN2, EPHA4, HP1BP3, MED14, HDHD2, GNB4, PCSK6, RPRD1B, AHR, RBPMS, CHMP2B, WAPL, IDS, TNKS2, GRIP1, RELL1, TAB2, POLR3G, SERP1, NF1, IMPAD1, MAP4K3, HOXA5, KDELR1, FOXN2, SLC9A2, FKBP5, ALG9, CUL3, PODXL, TRHDE, PTPN4, PROS1, C2orf69, MAST4, ULBP1, FOXO1, CDH20, HMGXB4, MAN1A2, TWSG1, STMN1, FAM13A, GRIN3A, ADGRF5, CCSER2, FOXP2, NKAIN1, RPE, NRN1, KDM7A, USP15, SLC31A1, FOXN3, SLC6A1, PTBP3, SERTAD4, PAX6, TNPO1, BBOF1, BCR, TWISTNB, MYT1L, ZBTB21, TCERG1, KLF5, ARHGAP29, PDE5A, PPP2R2A, THBS1, TXNRD1, KLHL18, PPP1CB, ERBIN, UNC13A, WDR44, KLHL31, ATP2B4, RAB22A, TARDBP, ARHGAP5, GRIK3, PDE10A, CACNB2, RHOQ, STXBP4, PAN3, PDHX, CLCN3, NABP1, LRP12, ZFYVE16, TRMT10A, TRPM3, TMEM64, MED12L, RNF139, BICD2, SHANK2, CHIC1, TAB3, HCN4, FAT1, C20orf194, CELSR2, HELZ, TET1, TTC14, SOCS5, BMI1, GNB1, SKIL, MKX, CRTC1, C16orf70, GSE1, VAMP7, BNC2, TTC33, SYNM, DEPDC4, DYNLT3, AAK1, CTTN, KTN1, ARSJ, MEF2C, CBFA2T3, PTGER3, KANK1, GPHN, ATRX, RYK, MBNL2, NID1, MSN, DIAPH2, PTPRZ1, DIP2C, ALKBH1, CD55, YOD1, TBC1D12, YIPF4, MRPL19, YTHDF3, MIER3, GOLPH3, ARF4, CCSER1, LARS, CHD6, ZFAND4, FAM135A, ARL4C, ADGRL2, DCUN1D3, CBR4, TP53INP2, OLFM1, RUNX1, PRKAA1, ENAH, ATP2C1, CROT, ESRRG, DLG3, PCDHA2, SSR3, RNF2, ADGRL1, PEAK1, PRR14L, SATB2, ZFHX4, MORC3, TMEM47, CHL1, PLEKHA3, UNC79, SMIM7, HOXA10, GABRB2, EGLN1, FAM167A, PARPBP, CRISPLD1, CAP1, DUSP1, CPM, TNKS, TMED2, PRUNE2, FCHSD2, PFKM, DNAJB6, ITPR2, MLLT3, NUP50, ARRDC4, MAP4K5, WDFY1, CTGF, HMGB2, MTX3, FAM120C, ZNF831, XPR1, PDPN, BTBD3, PRKAR2B, PPP1R2, PCDHA10, TBL1X, RBM12, RFWD3, INSIG2, TMEM65, CACNB4, STX17, USP6, ADGRB3, PCDH11Y, FAM198B, HIVEP2, ORC2, AKAP1, UBL3, DIP2B, TOX4, AXIN2, DENND1B, DCUN1D1, DCUN1D4, SDC1, FUT9, PTPN21, XPO1, LATS1, GRM5, AP3M1, LGI1, ADGRL3, NR5A2, IQSEC1, NAV1, PLAGL2, LRRC7, PPP4R3A, CNTNAP2, CDK5R1, EDEM1, CBLN4, C21orf91, SPRY3, FLRT2, CEBPA, BRINP2, C2CD2, PCDH9, LMO3, XKR6, CSNK1G3, TGFBR2, PCDHA1, TCF7L2, ZFHX3, FZD6, CDC42SE2, RNF130, ENC1, ZBTB44, ZMAT4, RLIM, KITLG, ARID4B, SETX, LHFPL3, DERL1, LMNA, FOXO3, WNK1, CSRNP2, SUPT7L, SERBP1, CRKL, ANP32B, AP4E1, DAAM1, RNF44, SAR1B, SCN9A, TOB1, CCDC88A, RAB7A, SPOCK1, RNGTT, KCNJ2, KLHL2, U2SURP, MITF, PAIP1, IGF2BP3, FNBP1L, SH3BP4, TRIM33, PIKFYVE, ATG2B, SHC1, ARL4A, ARHGAP21, HOOK3, CAND1, UTRN, PALLD, KPNA3, ANLN, BIRC5, PPP3CA, ZNF529, BMT2, GATA6, HECTD2, PRDM16, TRIM2, DMRT3, YWHAQ, STARD13, SON, RAB35, TMEM117, PRPS2, ABRAXAS2, NIPBL, RAB5B, MAPRE1, APPBP2, KIAA0355, CPEB1, KDM5A, ATF1, PALM2, OCRL, PNOC, HLF, PKIB, HOXD13, SLAIN1, AHCYL1, MTR, ASH1L, PRRX1, KLHL9, GNAO1, RPS6KA6, RPL10, SH3D19, OSBPL10, SCN3A, ASXL2, ZFPM2, MOSPD1, FRMPD4, YPEL5, AGO2, BVES, RBM26, KIT, ZNF827, EPHA7, ANTXR1, CORO1C, MCFD2, WNT5A, ATXN7, HAS2, DNMT3A, CNOT7, CCND1, FAM122A, DPY19L3, PLCXD3, DMXL1, SIK2, GRM1, CNTN5, LRIG1, ITGB8, ERC2, PCDH11X, RIMS1, UBE2K, XPOT, MFAP3L, BCL11B, ARID2, SMG1, TRMT11, FILIP1, ARGLU1, POLR2M, REEP1, CREM, SPAST, PRDM1, ALS2, EI24, GREM2, VASH2, BUB3, GFPT1, KPNA4, NECTIN1, RCOR1, YWHAG, TMEM68, FOXJ3, ZIC5, SASH1, PCDHA5, PIGH, GTF2H1, NAMPT, YAF2, KIDINS220, PLCB4, TSC1, MSH4, PPP1R14B, DMXL2, EID1, STIM2, RBM14, MAML1, ZNF706, ABHD13, CPSF6, ATF2, SYNCRIP, MED13, AFDN, SMPD3, CALN1, PCMTD1, PI4K2B, LHFP, SHOC2, SLC25A36, MAPRE2, LUC7L3, NACC1, SEC62, SP3, FLRT3, COL4A1, NAV3, ZCCHC11, SUCO, LAMP2, SPSB1, WDR48, NRXN3, PTGR2, RBM39, INPP4A, LARP4, ZMPSTE24, SHOX2, CERS6, EPAS1, MKRN1, NXT2, MORF4L1, GALNT1, ANKMY2, ARCN1, MACROD2, CYBRD1, TMEM26, SIK1, KAT7, NUDT21, PPM1D, PCDHA6, AP1AR, MOB3B, RLF, TOMM20, PTEN, SENP1, GPR180, VCPIP1, SSH2, EXOC5, GK, AK3, GPR3, MDGA2, NSD2, ZNF202, SLC23A2, PKD2, VPS26A, ZNF704, PAIP2, LRRC8C, PTPN11, FBN2, GDF6, DICER1, PCDHA4, CHST1, MFAP3, PCMT1, SOCS6, BTRC, LSM14A, SLC25A16, ABCA1, RBM4, ATP13A3, MYO5B, USP24, MYEF2, SYPL2, SGCB, RCOR3, KRAS, CNN3, PRDM4, HDAC9, RAB21, C12orf29, KIAA1549L, BICRAL, NR3C1, EMP1, MTOR, SLC2A8, STK38L, MORF4L2, SUB1, SNX4, SMAD7, TMEM57, PIP5K1A, REV3L, SPRED1, ADAM10, SPEN, NSD3, NAPEPLD, GALNT7, IL1RAP, TXNDC5, PER2, TAF4, ARHGAP19, TFRC, VPS13A, ILF3, SLC7A8, LAMC1, CLTC, ZBTB8A, PSME4, PCNX1, SEMA6A, CREB5, ICK, FAM60A, SEC23A, TPD52, PTPRK, MSL2, SLC11A2, DENND5A, ARHGEF37, DOCK9, DLG2, MARCKS, ZYG11B, PPARA, RORA, CPEB2, BPTF, EPB41L2, JMJD6, ELAVL4, SBF2, NFIB, LRCH2, TRIM13, ANKRD13C, ARRDC3, NIN, SPG21, LIMCH1, SCN2A, DOCK10, KLHL8, PRKD1, ANKRD50, RECK, ATPAF1, FBXO41, PCDH10, CELSR1, HDAC2, SLF2, GTDC1, TRIM39, TMEM170B, PRLR, ZFYVE26, DR1, CAB39, PCDHA3, MGAT4A, NAA30, ARID4A, CEP41, FYTTD1, ROBO2, ZMYND11, PDK1, OXSR1, MTSS1, TMEM30A, PDS5A, NEDD9, C6orf106, CUL4A, CDC42BPB, NPY2R, BMPR1B, SEMA5A, PDSS2, C18orf54, ATP2A2, LGR4, C3orf38, SCAF11, FKBP1A, FCHO2, AMD1, RETREG1, DNAJC13, FRMD4B, DNAJC10* |
| 1 4 | 445 | *ZHX3, EP300, DTNBP1, ZEB1, MMS22L, MAPKAPK2, TMEM19, PDCD4, CTDSP2, ETNK2, HDAC7, ADH5, RFX3, PIRT, BCL2, SLITRK4, TM6SF1, TFAP2B, TSN, DNHD1, LONRF2, UBE2W, FHL1, TRMT6, RSU1, CAMSAP1, NRF1, PLSCR4, CADPS2, JPH2, TRAM2, PI15, RCAN1, UPK1B, NTN4, SEPHS1, TRIM56, NR1D2, STX5, WSB1, ACTRT3, NRXN1, UNKL, KIF1B, AMOTL2, DUSP10, LRRFIP1, CBX2, GRID1, ACER3, SLC22A5, UNC119B, C9orf72, ST7L, SLC30A8, CDK13, PRC1, LNPK, LPIN2, ZHX2, SLITRK3, EML4, IPCEF1, PRDM10, CALCR, ATXN1L, GPAM, ZER1, SNX22, POLH, PDE4DIP, COL4A6, ZMYM4, DDHD1, TMEM41A, C3orf58, SLC39A10, RAB11FIP5, SPIN3, RASA1, GJC1, PIK3R1, SV2A, NEXMIF, ATG12, ZHX1, KDM2B, RAB10, ARHGAP24, N4BP2L1, KCTD3, NKX2-2, DNAJB14, XKR4, KAZN, PAX5, ARL5A, PHKB, ZNF697, TRIM5, ZBTB40, ZNF445, SRSF12, ZADH2, ACTR3, EIF4EBP2, TFDP2, FOXQ1, NDST1, C5orf24, SH3RF3, ABL2, TIMP3, CCR9, TBX5, MRVI1, HTR2C, PGRMC2, RAP1GAP2, DDAH1, PPP1R8, SPRTN, FAM133B, FGD4, LRRC4, ETS2, BAG1, PAN2, GLB1L, MAPK9, PTGFR, TRIM37, GRHL2, SELE, FAM171A1, PLAGL1, FLOT2, LEPR, LTBP2, ADAMTS19, LARP1, DBT, EXOC8, EDNRB, PPP2R5C, SYNJ2, WDR36, ADAM9, PKP4, ATXN3, TRIM27, RABGAP1, IER5L, MRC2, LTN1, ZCCHC3, LRRC15, CSE1L, SMAD4, RIOK3, ATG9A, MIGA1, CCNT2, ZBTB6, PRR11, PTAR1, KIF3B, SNX7, POLR2D, FXR1, CASP3, FBXO21, P4HA2, SMC2, LATS2, MS4A1, EZH1, NUS1, PTPRN2, SH3BP5L, KLF7, UBE2R2, SNX27, FOXO4, CERS5, EIF5, ERBB3, NUP58, SLC1A1, SLC6A19, RNF144B, ZNF460, SPTB, STRBP, SGMS2, IBTK, CDC73, FAM43A, ZNF280B, FBXW11, MAP9, ANKRD12, CD96, TMOD1, METTL4, PPP1R16B, COBL, KLF15, VANGL1, NRCAM, DDX58, FAM107A, CAMKK2, EGLN3, SREK1, SHC4, CBX5, ZNF662, ZNF592, KPNB1, C1orf116, FBXO32, DHX36, APPL1, NDC1, RASSF1, AARS, PAK2, WASF2, MDM4, MAMLD1, PRR3, ATRN, PAK1, CACNA2D1, RAB3IP, FADS1, Sep-14, CDC14A, CPLX3, BCLAF1, SLC6A15, OTUD4, SORT1, RBFOX1, SCP2, ZNF605, ADAM19, MICU1, PIK3AP1, SUV39H2, SLC38A4, PKHD1, UBXN7, FRYL, FN1, CSRP3, FAM126B, ANKRD52, GABBR1, CAPN6, DIXDC1, C1orf198, NSD1, DSTYK, SLC39A9, ATG7, IL13RA1, PTGS2, IMPACT, SMCO4, CD79B, PPM1E, UBE2L3, MCTP2, FIGN, KCNIP1, KIAA0087, PDK4, GABRA1, MYOCD, PDE7B, B3GALT2, UBR1, COL9A1, FEM1C, IL17RD, HECTD1, GDNF, ATP9A, PRIMA1, TRIM66, TTC39A, SLAIN2, CBX7, RALGPS2, GSPT1, ERLIN1, FBXW7, RIC8B, UBE2Z, RPS6KA1, USP36, PPP1R11, S100PBP, MESD, MRPS25, MRPL42, SIRT1, UBFD1, WIPF1, PIP5K1B, RNF8, KLF13, DNAL1, POU2F1, CNNM2, SLC39A1, AUTS2, PBX2, INSIG1, CACNA1G, AMER1, PDLIM5, GOLPH3L, B3GNT2, DUSP13, MYADM, PGK1, SLC30A9, MEF2D, MYO1B, BTLA, TXNDC15, NETO2, SYNPO2, KCTD15, PTPDC1, KDELC2, NRP1, PHLPP2, BASP1, CALML4, AMN1, YWHAE, BDNF, SEMA3A, SLC2A3, SPOPL, DHRSX, DHX33, TSC22D3, CPSF7, PTPRB, ACTR1A, MMAB, TTBK2, C8orf37, VAPA, FSTL4, PFN2, AGO4, FAM222B, USP13, VAPB, TUFT1, PRKAB2, GATAD1, ITGAV, PPP2CB, CD2AP, MYO5A, AGPAT4, FAM53C, DIEXF, ACO1, PFKFB3, SPECC1, DPY19L2, RRBP1, ERI2, PLEKHH2, PTCH1, GRIA3, HNRNPH3, FAF2, CLASP2, PDE1A, LMTK2, KCNJ14, TMSB4Y, GOLGA7, PHLDB1, CLOCK, KCNK2, HSPA13, NFASC, DGKG, RASGRP1, KREMEN1, SNAP23, ZDHHC9, ZNF451, ZNF322, H6PD, ENSA, ZNF449, TMEM245, Sep-11, PAFAH1B2, SIDT2, JPT2, HIF1AN, USP48, SRSF2, CLVS1, FMO2, ATAT1, MLEC, RGS6, GXYLT1, CYB561D1, PKDCC, KSR2, TAPT1, TTPAL, FAM160B2, MFSD5, DUSP16, ELL2, FZD1, DCTN5, NUP43, THAP6, NANOS1, NRG1, SYT9, TAOK1, CASK, GIT2, ATP10B, GGA2, SLC10A3, USP3, RPS6KB1* |
| 2 3 | 23 | *CXCR4, IL20RA, CD83, ASF1A, SPRED2, ZFP36L2, STAT3, RNFT1, SDK1, DDX6, KIF21B, IL22RA2, TMEM25, PITPNM2, TSPAN31, NCOA5, MAPK1, B4GALT5, SATB1, CENPO, USP34, ETS1, TRIB2* |
| 2 4 | 24 | *TUBD1, MFN2, AHI1, VMP1, BANF1, PAPD7, PCNP, TM9SF2, LBH, ZBTB38, STAT4, CAMK2G, PTPN1, TXK, NDST2, IQGAP1, PDE4C, POGLUT1, PLAU, PLXNC1, CBLB, CUX2, CD80, GGNBP2* |
| 3 4 | 367 | *CD44, BTG2, CDK14, CHD9, RPS6KA5, ITGA3, EIF4G3, POU3F1, COL1A1, ACBD3, ARHGEF9, ST8SIA4, MARF1, THRB, SEC23IP, SRP72, THAP1, KDM2A, PDP1, NMNAT2, SH3PXD2A, KRCC1, GPBP1L1, PPARGC1A, CUL4B, AKIRIN1, HOXB8, RPN1, TANC2, KIF4A, SUSD6, KALRN, NEGR1, PARD6G, KDM6B, FBXO3, YPEL1, VEGFA, GIMAP6, AHCTF1, ITGA2, C6orf120, SPINK13, PPP4R1, ZC3H14, SETD3, STK39, TRIM6, AMPH, SIAH2, KMT2D, GIPC2, SH3TC2, WDFY3, C5orf51, CDON, NLK, PCDH20, KLHL15, NBEA, CHML, TMX3, TRAK2, NEO1, STAT5B, ADGRG2, GLIS3, ABCC5, MBTD1, USP33, CMTM4, MCC, NSFL1C, MBNL3, SYNJ2BP, RAP2A, KBTBD2, PTGFRN, RNF10, EVA1A, ITCH, SBSPON, PTPRJ, TCF20, KLHL5, OTULIN, SH3RF1, FAM46C, UST, GRAMD2B, ALMS1, NCOA7, C2orf88, FZD4, LYRM7, C15orf54, SAMD4B, HNRNPU, ATXN2L, NEMP1, XPO7, ZNF280D, YPEL2, PNP, B3GNT5, FAM234B, SMARCC1, TSPAN3, BMPR1A, STAG2, FUBP3, ATP11A, LRRC58, RAB14, BCL2L2, AQP4, COLGALT2, DSC2, RNF103, GRSF1, MSI1, ARPC5, SV2B, PITPNA, RIF1, SAMD8, SLC38A2, PRRC2C, EREG, ATG13, GOSR1, WASF3, TRAPPC10, GTF3C2, KCTD9, OPALIN, GABPA, USP37, HNRNPC, RNF165, YWHAB, H2AFJ, PAPD5, MGAT2, SYT6, BCORL1, ATP6V1H, TDG, SECISBP2L, COMMD10, FAR1, PURG, CEBPG, RNASEL, RETREG3, GCFC2, GLYR1, SMU1, CASZ1, OTUD7B, SLC30A5, IYD, C3orf18, C18orf25, RABL3, HERC4, SLC38A1, SYNJ1, PTBP2, CANX, DESI2, ADIPOR2, ZNF462, Mar-06, ZNF28, VOPP1, PPP2R5E, FEZ2, CCDC89, SCN2B, MEX3C, KPNA6, QTRT2, VTA1, E2F3, WAC, DPP10, CELF4, MIB1, ATG14, PITPNB, DPYSL3, ATP2B1, PUM1, DCAF8, DLG1, RNF38, SLC16A14, TET3, ARF6, ZNF280C, ZNF25, ABHD2, ALCAM, TMEM33, ITPRIPL2, SERTAD2, PGM2L1, MEIS1, AMMECR1, SGK1, DNAJC16, NAPB, TRAPPC2, ZNF518B, SPAG9, EPS15, CTNNB1, COL19A1, ZNF740, IPO5, GIGYF2, HIPK3, CEP68, SAR1A, NLGN3, LMX1A, GLCE, PIGK, SLC2A1, SMAP1, SYP, GLCCI1, ZFX, GPR155, MYBL1, MDGA1, ADARB1, GPC4, HNRNPD, GAREM1, IGF2BP2, CNTLN, PHC1, HDAC4, FYB1, RCHY1, DCAF6, SPOP, RPGRIP1L, AKAP6, FLG2, SOGA3, SORCS3, BAZ2B, CEMIP, PLEKHG4B, SHB, KCNJ3, ZDHHC13, VASN, SSR1, GABRB3, ADAMTS3, SMG7, ENTPD1, PURB, ZNF512B, SLC7A1, SNX15, COLGALT1, CEACAM1, RBM25, LRAT, FBXO28, EPHB2, ZC3H12B, RUFY2, RANBP9, CAPZB, ERC1, KAT2B, SLC6A17, KHNYN, SMNDC1, CTNND2, FRMD3, AGFG1, SETD2, TIA1, HMGB3, RC3H1, SOX21, RNF141, DACH1, JADE3, LPGAT1, CDKN1B, BCLAF3, FOXD3, GCM2, ARF1, RHOA, ZNF138, CRY1, PHOX2B, PIK3R3, FAM169A, SLC19A3, ZIC1, SOX12, PDPK1, MYCT1, FAM168B, CCSAP, CRIM1, ZNF644, TULP4, MEGF9, OTP, MAN2A1, PTPRT, GID8, CMTM6, C16orf52, MAP3K1, UBE4A, CCDC68, EIF4B, KATNBL1, VEZF1, EPB41, NUP98, ZFP91, ZNF229, MGA, SPTLC2, KMT5B, LEPROTL1, PPP2R1A, EPG5, ERO1A, ZNF367, HSP90B1, KIF1C, PTPRA, METTL7A, PA2G4, CYP26B1, YLPM1, KIAA0319, HEY2, ETV5, HNRNPA3, RHOBTB3, TMEM55A, POFUT1, ARF3, BLOC1S6, ZZZ3, RPF1, IGFBP4, CEPT1, BCAT1, ZNF33A, PGPEP1, CTSC* |

EV – extracellular vesicle; miRNA – microRNA; MS – Multiple Sclerosis.
